# Sociodemographic markers of high Sensory Processing Sensitivity: a descriptive study

**DOI:** 10.3389/fpsyg.2025.1617089

**Published:** 2025-07-08

**Authors:** María-Luz Morales-Botello, Moisés Betancort, Manuela Pérez-Chacón, Rosa-María Rodríguez-Jiménez, Antonio Chacón

**Affiliations:** ^1^Department of Computing and Technology, School of Architecture, Engineering, Science and Computing – STEAM, Universidad Europea de Madrid, Villaviciosa de Odón, Spain; ^2^Department of Clinical Psychology, Psychobiology and Methodology, Universidad de La Laguna, La Laguna, Spain; ^3^Spanish Association of Highly Sensitive Professionals and Psychologists, PAS España, Madrid, Spain; ^4^Department of Psychology, Faculty of Education and Psychology, Universidad Francisco de Vitoria, Pozuelo de Alarcón, Spain

**Keywords:** Sensory Processing Sensitivity, sociodemographic characteristics, personality trait, sensitivity levels, wellbeing, health, descriptive study

## Abstract

**Introduction:**

Sensory Processing Sensitivity (SPS) represents a personality trait characterised by heightened responsiveness to environmental stimuli, which can lead to both beneficial and adverse outcomes. Despite the exponential growth in knowledge about SPS in recent years, sociodemographic dimension related to this trait remains under-researched. The primary aim of this study was to analyse and provide deeper insights into the sociodemographic characteristics that may distinguish highly sensitive individuals. The present study was approached from the perspective of different sensitivity levels (low-SPS, medium-SPS and high-SPS).

**Methods:**

To examine the sociodemographic expression of SPS, we pursued two main objectives. Firstly, by logistic regression analysis, we investigated the sociodemographic characteristics that predict high-SPS. Secondly, by analysis of variance and post hoc analysis, we investigated whether the relationship between SPS and sociodemographic variables depended on the SPS level. We conducted these analyses based on a large sample from the general population (9,447 participants were initially considered).

**Results:**

The logistic regression analysis identified significant predictors of high sensitivity, spanning demographic, social, and wellbeing-related variables. Specifically, gender, age, civil status, number of children and type of residence as demographic variables; number of social groups and satisfaction with partner as social variables; and practise of body awareness activities as a wellbeing variable significantly predicted high-SPS. Moreover, analysis of variance and post hoc analysis, evidenced that unlike low-SPS and medium-SPS, high-SPS (SPS trait) was relatively stable with respect to sociodemographic changes.

**Discussion:**

We discuss our findings within the context of SPS, personality traits, and their practical implications for clinical, educational, and occupational settings. We hope that this work will contribute to identifying those who may need greater support in developing their wellbeing.

## Introduction

Highly sensitive people (HSP) or individuals with a high level of Sensory Processing Sensitivity (SPS) show deeper information processing and tend to be much more sensitive to environmental influences (Aron and Aron, 1997; Lionetti et al., 2018). Numerous studies in both adults and children associate SPS with a higher probability of negative clinical outcomes such as anxiety, depression, stress, and physical symptoms (Ahadi and Basharpoor, 2010; Bakker and Moulding, 2012).

Based on these implications of the trait and the relatively high proportion of high-SPS individuals, around 20–35% (Aron and Aron, 1997; Pluess et al., 2018), it would be very informative to know if there is any sociodemographic profile associated with this personality trait. However, there remains a lack of systematic research into the sociodemographic characteristics associated with SPS. Furthermore, although the SPS construct has been widely studied since its introduction in 1997 (Aron and Aron, 1997), certain important issues related to the SPS trait, such as its manifestation throughout the life cycle, have not yet been completely clarified.

The present work focuses on covering the two important gaps described above, for which a general objective is proposed: working with a large sample from the general population, this study aims to analyse and elucidate sociodemographic characteristics that may serve as potential differentiators of highly sensitive individuals.

### Sensory Processing Sensitivity construct

The SPS construct was initially introduced by Aron and Aron (1997) in a study that examined this basic individual difference and its relationship to introversion and emotionality, characteristics with which this individual difference had been confused in previous personality theories. This and many other subsequent studies have analysed similarities and differences between SPS and other constructs or personality traits (Greven et al., 2019), and although these analyses have shown some overlap with other constructs (Hellwig and Roth, 2021), there is no complete overlap, and SPS is conceived as a conceptually different construct (Greven et al., 2019; Pluess et al., 2018; Smolewska et al., 2006).

SPS is measured using the Highly Sensitive Person Scale (HSPS). Since its original unidimensional 27-item version (Aron and Aron, 1997), numerous cross-cultural adaptations and validations have been made, as well as a reduced versions for adults, adolescents and children (see, e.g., Baryła-Matejczuk et al., 2022; Weyn et al., 2021, 2022b). The most repeated factor structure is what has recently been proposed as a bifactor model: global scale and three subfactors (ease of excitation (EOE), low sensory threshold (LST) and aesthetic sensitivity (AES)). Nevertheless, although this is the most common subfactor structure, other studies have led to a variety of factor solutions, ranging from 1 to 5 (Aron and Aron, 1997; Chacón et al., 2021; Ershova et al., 2018; Sengül-Inal et al., 2018; Smolewska et al., 2006). Furthermore, a recent study has found a six subfactor structure in a 43-item instrument proposed to measure SPS (De Gucht et al., 2022).

### Theoretical framework of Sensory Processing Sensitivity

Biological Sensitivity to Context (BSC), Differential Susceptibility and Sensory Processing Sensitivity (SPS), are integral components of the theoretical framework of environmental sensitivity (Greven et al., 2019). BSC, conceptualised from a physiological perspective, is defined as neurobiological susceptibility to both positive and negative environments, mediated by increased activity in stress response systems. This theory originates from empirical observations of differences in autonomic and adrenocortical reactivity in children exposed to risk situations (Boyce and Ellis, 2005). In parallel, Differential Susceptibility frames individual differences in sensitivity from an evolutionary perspective, representing diverse developmental strategies (low or high plasticity and adaptation) maintained by natural selection (Belsky and Pluess, 2009). Both theories converge in conceptualising reactivity to environmental stimuli as an indicator of sensitivity or susceptibility to environmental influences (Ellis and Boyce, 2011).

The SPS theory introduces a psychological and personality component to individual differences in sensitivity. Specifically, it is based on the integration of animal studies and other personality/temperament theories concerning behavioural inhibition, shyness, and introversion in children and adults (Aron and Aron, 1997). The SPS trait is proposed to be a phenotypic trait with a genetic-base and environmental modulation. The theory is further embedded within the overarching framework of environmental sensitivity (Pluess, 2015).

All these theories concur that individuals vary in their sensitivity to both negative and positive environments. Genetic and environmental components, as well as their interaction, may be shaping the development of individual differences, making some individuals more sensitive than others. Therefore, in this study, we considered that genetic and environmental differences in environmental sensitivity may have a sociodemographic manifestation, and in-depth description of this manifestation is the core of the present work.

### Importance of studying sociodemographic characteristics of the Sensory Processing Sensitivity trait

Over the last 20 years, a very high number of studies have linked the SPS trait with an increased probability of experiencing adverse clinical outcomes in both adults and children, encompassing both physical and psychological health issues, including behavioural problems and difficulties in relationships with others (Attary and Ghazizadeh, 2021; Benham, 2006; Pérez-Chacón et al., 2021).

#### Physical health

Through general physical health or physical symptoms questionnaires, numerous studies have consistently demonstrated that the SPS trait is associated with poorer physical health and a higher number of self-perceived physical symptoms, found from direct correlations with SPSQ (De Gucht et al., 2022), HSPS (Benham, 2006; Kenemore et al., 2023; Le et al., 2020) and through correlations with some SPS factors, especially with EOE and LST (Ahadi and Basharpoor, 2010). Recent research has focused on studying more specific physical complaints. In this sense, increased likelihood of experiencing a gamut of gastrointestinal symptoms (Iimura and Takasugi, 2022) and greater listening-related fatigue (McGarrigle and Mattys, 2023) have been reported in HSP. Furthermore, it has been found that migraine with aura patients had a significantly higher score for LST in comparison with control patients (Rajić et al., 2023). Also, higher SPS levels and a significantly higher frequency of SPS trait were found in a group of adolescents with type 1 diabetes compared with a control group (Goldberg et al., 2018). Another recent study in adolescents has shown some relationship between SPS and chronic pain. Although a global score on the SPS scale was only marginally associated with pain intensity, the proportion of high-SPS individuals was large (45.68%), and high-SPS was predictive of health-related quality of life, with lower scores for physical, emotional, and school functioning subscales (Koechlin et al., 2023). Interestingly, clinically, parents of children with atopic dermatitis often give the impression of increased sensitivity. One study has examined whether these parents showed characteristics of SPS trait (Liffler et al., 2019). The results revealed higher sensitivity and excitability, reduced tolerance to frustration, more depressed mood, lower life satisfaction and increased stress in atopically predisposed parents.

#### Psychological health

High-SPS has also been consistently associated with increased anxiety (Bakker and Moulding, 2012; De Gucht et al., 2022; Jakobson and Rigby, 2021; Licht et al., 2020; Neal et al., 2002; Panagiotidi et al., 2020; Takahashi et al., 2020), depression (Bakker and Moulding, 2012; Drndarević et al., 2021; Hoffmann et al., 2022; Jakobson and Rigby, 2021; Le et al., 2020; Panagiotidi et al., 2020; Wu et al., 2021; Yano et al., 2019), or stress (Bakker and Moulding, 2012; Benham, 2006; Hoffmann et al., 2022; Jakobson and Rigby, 2021; Panagiotidi et al., 2020; Rubaltelli et al., 2018; Weyn et al., 2022a; Wu et al., 2021). Likewise, some studies with young adults have found a positive correlation of EOE and LST with psychosomatic symptoms (Takahashi et al., 2020) and with psychological health complaints (Listou Grimen and Diseth, 2016) and a negative correlation with wellbeing measures (Takahashi et al., 2020). It has also been found that the SPS trait is related to other disorders. For example, individuals with Seasonal Affective Disorder (SAD) exhibited higher SPS levels compared with control healthy individuals. The prevalence of high-SPS was 5 times higher in individuals with SAD, and high-SPS was associated with greater severity of SAD symptoms (Hjordt and Stenbæk, 2019). A positive correlation has been reported between high-SPS and avoidant personality disorder (Meyer and Carver, 2000) a generalised subtype of social anxiety disorder (Hofmann and Bitran, 2007), and attention deficit hyperactivity disorder (Panagiotidi et al., 2020), as well as moderately strong, positive relationships between SPS and characteristics of autism (Attary and Ghazizadeh, 2021; Liss et al., 2008) and alexithymia (Attary and Ghazizadeh, 2021; Jakobson and Rigby, 2021; Karaca Dinç et al., 2021; Liss et al., 2008; McQuarrie et al., 2023).

Additionally, the SPS trait has also been studied in work environment contexts. Vander Elst et al. (2019) reported a greater susceptibility to the work environment among HSP. Specifically, EOE and LST amplified the relationship between job demands (workload and emotional demands) and emotional exhaustion, and LST also strengthened the relationship between job resources (task autonomy and social support) and helping behaviour. Subsequent studies have linked SPS with worse quality of professional life at the level of burnout and compassion fatigue (Chacón et al., 2023; Golonka and Gulla, 2021; Meyerson et al., 2020; Pérez-Chacón et al., 2021).

Finally, SPS has also been found to be an independent risk factor for developing negative outcomes in romantic relationships (Zorlular and Uzer, 2023).

#### Contextualising the impact of SPS

In the previous sections, considerable evidence that directly relates the SPS trait to negative consequences for people's health, behaviour or relationships has been presented. This section builds upon the prior review by incorporating additional studies of SPS as a moderating/mediating variable, and studies that contextualise the impact of SPS under certain environmental conditions. These environmental conditions can include any salient conditioned or unconditioned internal or external stimuli, including physical environment (e.g. food, caffeine intake), social environment (e.g. childhood experiences, other people's mood, crowds), sensory environment (e.g. auditory, visual, tactile, olfactory), and internal events (e.g. thoughts, feelings, bodily sensations such as hunger or pain) (Greven et al., 2019).

The role of SPS as a moderating/mediating variable has been highlighted for several decades. For example, the moderating role of SPS on the relationship between pessimism and avoidant personality disorder (APD) is known (Meyer and Carver, 2000). This study suggested that pessimism is more strongly related to APD features among HSP or those who recall adverse childhood experiences (e.g. isolation, rejection, conflict). SPS also acts as a mediator of the relationship between childhood trauma and adult psychopathology (Karaca Dinç et al., 2021) and between childhood experiences and life satisfaction (Booth et al., 2015). In addition, a mediating role of SPS is found on the relationship between attachment anxiety and physical symptoms (Le et al., 2020), as well as on the impact of stress in depressive symptoms (Wu et al., 2021). All the above exemplifies the importance of environmental conditions during childhood in highly sensitive people and the distinct sensitivity types that could emerge due to different developmental conditions (Bürger et al., 2024; Huang and Pluess, 2025; Pluess, 2015).

In addition, other factors potentially moderating/mediating the negative impact on high-SPS individuals have been investigated. A study that examined how mindfulness and acceptance can moderate the relationship between SPS and distress revealed that while SPS was related to higher levels of depression, anxiety and stress, SPS was only related to anxiety when mindfulness and acceptance were low (Bakker and Moulding, 2012). The construct “sense of coherence” is evinced as a moderating element of the relationship between SPS and depressive symptoms (Yano et al., 2019). Takahashi et al. (2020) also found a mediation of dispositional mindfulness in the relationship between SPS and anxiety, wellbeing, and psychosomatic symptoms.

In contrast to the numerous negative outcomes related to high-SPS, many positive, even advantageous aspects have been described as well. For instance, better response to psychological interventions (Kibe et al., 2020; Nicolson et al., 2022; Pluess and Boniwell, 2015), or increased susceptibility to positive experiences and exposures, and to environmental quality (Iimura, 2021; Pluess, 2017). Overall, due to differential susceptibility and environmental sensitivity, high-SPS individuals are more affected by their environments, both for better and for worse. However, even though high-SPS can be advantageous under certain conditions, the evident risk of developing a range of serious health problems due to increased environmental sensitivity suggests that this group requires attention.

### Sociodemographic factors in relation to personality and health

The sociodemographic factors-personality-health triads have emerged as a significant focus of research in recent years. For instance, Løset and von Soest (2023) examined whether personality is associated with sickness absence, and whether health and sociodemographic factors (gender, age, type of occupation and job satisfaction) moderate this relationship. The results pointed towards a moderating effect of age and type of occupation on the relationship between personality and sickness absence. Specifically, a significant interaction effect of age and openness on sick leave was found, indicating that openness increased the risk of sick leave for older employees compared to younger employees. From a mechanistic perspective, they argued that probably for younger people, openness may promote adaptation to shifting work demands and integration in new workplaces which are of importance early in an occupational career. Conversely, for older people, high openness may hinder job performance and increase sick leave rates when extensive experience makes work less challenging and more monotonous. Furthermore, these authors proposed as an interesting area of future research the study of how sociodemographic factors and work-related factors moderate the relationship between personality and sickness absence. Willroth et al. (2022) analysed emotional responses to a global stressor and showed that individuals differ substantially around the average emotional trajectories and these individual differences were predicted by sociodemographic characteristics and stressor exposure. They also discussed how identifying predictors of individual differences can inform who is in greatest need of societal support, as well as the specific risk and protective factors that may be useful targets of interventions to promote emotional wellbeing. Moreover, specific aspects of the psycho-emotional domain, which strongly impact health and are closely related to personality traits such as negative emotionality and emotional dysregulation, have also been studied, including demographic and social perspectives (Bianchi et al., 2022; Geng et al., 2020).

### Sociodemographic factors and Sensory Processing Sensitivity

#### Demographic factors

A number of prior studies regarding Sensory Processing Sensitivity research have reported SPS scores segregated by gender, with the majority pointing to significantly higher total SPS scores among women [Drndarević et al., 2021; Iimura, 2022; Karaca Dinç et al., 2021; Meyer and Carver, 2000; Panagiotidi et al., 2020; Pluess et al., 2023; Sengül-Inal et al., 2018; Setti et al., 2022 (Study 1); Wang et al., 2022], as well as on some or all SPS factors (Chacón et al., 2021; Jentsch et al., 2022; Licht et al., 2020; Pérez-Chacón et al., 2023). In contrast to the coherence observed in gender-related findings, research concerning SPS and age exhibits certain inconsistencies. For instance, research conducted with children, adolescents and young adults has yielded inconsistent results, with some studies reporting no significant correlation between age and SPS [Schmitt, 2022 (Study 1); Setti et al., 2022; Sperati et al., 2024], while others demonstrate either positive (Baryła-Matejczuk et al., 2022; Costa-López et al., 2022) or negative relationships (Licht et al., 2020; McGarrigle et al., 2021; McGarrigle and Mattys, 2023; Meyerson et al., 2020; Panagiotidi et al., 2020). On the other hand, a study conducted with adults examined age-related changes in the three dimensions of SPS (Ueno et al., 2019). Results indicated that LST and EOE decrease linearly with age, whereas AES increases linearly with age. In addition, age-related changes in Sensory Processing Sensitivity do not differ by gender.

#### Other sociodemographic factors: education level, culture and professional sector

Education level is a key socioeconomic factor across multiple domains, including psychology. However, its association with SPS remains understudied, and existing research shows inconsistent results—positive correlations (De Gucht et al., 2022; Pieroni et al., 2024) vs. negative [Setti et al., 2022 (Study 1)] or non-significant correlations [Setti et al., 2022 (Study 2)],. Regarding culture and professional sector, a study investigating neural activation patterns in Americans and East Asians found no significant differences in SPS in these culturally different groups (Aron et al., 2010) and professional sector has emerged as a factor contributing to significant differences in SPS (Chacón et al., 2023).

### The present research

The preceding sections have thoroughly outlined the significance of examining sociodemographic aspects within the framework of Sensory Processing Sensitivity (SPS), given its implications for health. Additionally, an emerging area of research linking sociodemographic factors with health and personality has been introduced. Finally, a review of the specific literature on sociodemographic factors and SPS has revealed inconsistencies, while also highlighting a relationship between sensitivity and sociodemographic charasteristics. Furthermore, certain demographic variables, such as the number of children or marital status, remain under-researched in this context, despite their potential to moderate or reflect SPS levels. Drawing upon a mechanistic approach and consistent with the theoretical foundations of SPS, it is plausible that environmental factors, such as parenthood (Morales-Botello et al., 2026; Sperati et al., 2025), may impact stress levels, stimulation, and physical, mental, or emotional load, thereby potentially elevating SPS levels. Regarding marital status, romantic relationships and cohabitation could impact individuals' SPS levels from a cognitive-emotional standpoint. Conversely, sensitivity levels, particularly high SPS, might drive life choices, such us living with a partner, marrying, divorcing/separating, or having children. This potential influence of SPS on individual demographic outcomes could be interpreted both as a behavioural adaptation to regulate environmental stimulation and as a consequence of the cognitive and emotional characteristics inherent to the highly sensitive personality trait.

Moreover, certain social variables remain under-researched within the framework of Environmental Sensitivity, and particularly in Sensory Processing Sensitivity. For instance, the variable “number of social groups” has been demonstrated to significantly impact quality of life and mortality rates, with particularly notable effects observed during the transition to retirement (Steffens et al., 2016), a period often marked by diminished social connexions. This variable may hold particular relevance in the context of SPS, as high sensitivity has been associated with inhibitory behavioural patterns, including withdrawal or social avoidance as a strategy to prevent emotional overload stemming from the characteristic heightened emotional and cognitive reactivity. Therefore, it would be valuable to quantitatively examine whether high SPS manifests differently in terms of social group participation and associated satisfaction levels when compared to low or medium SPS groups. Similarly, it is also expected that the level of satisfaction with a partner or colleagues may be related to sensitivity levels. This idea is supported by previous theories and research on SPS, which suggest that greater awareness and responsiveness to others' moods and emotions are central characteristics of high sensitivity, as well as, stronger emotional reactivity and deeper information processing (Acevedo et al., 2014). This way of experiencing relationships could manifest in terms of satisfaction with them, making these variables relevant within the possible sociodemographic characterisation of SPS.

Thus, the present research carries out a descriptive study of the SPS personality trait in sociodemographic terms, employing a retrospective ex post facto design (Montero and León, 2002). Our main aim was to analyse and gain a deeper insight into the sociodemographic characteristics that may differentiate highly sensitive individuals. Accordingly, we addressed two specific aims: (1) to investigate possible sociodemographic characteristics related to high-SPS and (2) to contextualise the SPS construct from the interaction between levels of sensitivity and sociodemographic variables. While prior research has shown a link between demographic factors and SPS, none has comprehensively explored this relationship in sociodemographic terms. This study was performed using the framework of three sensitivity levels (low-SPS, medium-SPS, and high-SPS) previously identified in the literature (Lionetti et al., 2018; Pluess et al., 2018) and was based on a large sample from the general population. Thus, two research questions motivated this study:

RQ1 What social and demographic variables are important in predicting high-SPS?
RQ2 Is the relationship between sociodemographic variables and SPS dependent on the sensitivity level?

RQ1 is answered from aim 1 and RQ2 through aim 2. To address aim 1, a logistic regression was conducted to identify the sociodemographic variables with the greatest weight in shaping high-SPS. To address aim 2, we conducted robust ANOVAs and post hoc analyses to investigate the possible interaction between sensitivity levels and sociodemographic variables for each SPS factor and to explore that interaction. Finally, consistent with the literature reviewed above, it is expected that the sociodemographic variables inform about SPS, although probably in relationships without a large effect size.

Knowing how the SPS trait is manifested through sociodemographic characteristics can be of utmost importance in identifying groups most vulnerable to the negative effects of the environment, due to their increased environmental sensitivity. The early identification of these groups to be able to promote both personal and environmental care could lead to a reduction of the negative clinical outcomes that may arise from the SPS trait, as well as a strengthening of its more advantageous aspects. Through objectives 1 and 2, this research enhances our understanding of SPS by examining sociodemographic characteristics.

## Methods

### Participants

The inclusion criteria were being resident of Spain and being at least 18 years of age. Under these criteria, 9,447 people from the general Spanish population enrolled in the study. The age distribution was as follows: 32.29% (18–24 years), 24.9% (25–34 years), 21.26% (35–44 years), 15.41% (45–54 years), 5.06% (55–64 years), 1.02% (65–74 years) and 0.07% (older than 74 years). Regarding gender, 70.64% identified as women, 0.20% as transgender (trans) women, 27.16% as men, 0.27% as trans men, 1.13% as nonbinary and 0.60% as other gender. For analytical purposes, individuals identifying as women or trans women were classified as female and participants identified as men or trans men were considered male; Master and PhD categories were grouped into a single category; and non-binary/others gender, age over 65 years, without education, and retirees were discarded due to low recruitment.

In addition, embedded in the perspective of three sensitivity levels, in order to enhance the precision of sociodemographic differentiation between SPS levels: (i) participants with a HSPS score in the medium-to-high sensitivity transition were excluded for the logistic regression analysis, resulting in a sample of 7616 participants; (ii) for the ANOVA and post hoc analyses, we additionally excluded participants in the low-to-medium sensitivity transition, resulting in a sample of 6507 participants.

Finally, given that our objective was to establish sociodemographic profiles, we used G-Power to calculate the representative sample size. We considered a demanding sampling scenario comparing means through a two-factor ANOVA (SPS levels and the sociodemographic variable with more categories) with a small effect size (0.10), high power (0.9) and an error alpha of 0.01. The program generated a total sample value of 3138 (approximately 175 per group). Additionally, we multiplied this sample size by 2 to improve the representativity of the sample.

### Procedure

This study used convenience sampling, collecting data via an online questionnaire on the Spanish Association of High Sensitivity Professionals' website (https://pasespana.org/). This association is well-known in Spain and Latin America, enhancing the dissemination and recruitment of participants with varied sociodemographic characteristics. Additional recruitment efforts were made through various channels within the institution conducting the study.

The questionnaire, implemented with Google Forms and Gravity Forms, took about 20 min and consisted of 11 steps. Participants first read a summary of the study, including details on voluntary participation, data anonymisation, the option to receive study results, and encouragement to answer honestly. Participants had to accept the informed consent, before beginning to answer the questionnaire. The complete questionnaire also included other measures not relevant to this study.

No financial compensation was provided to participants for their involvement in the study. The data was collected between the end of October 2021 and the middle of March 2022. The study received ethical approval from the Institutional Research Ethics Committee of the university where the research was conducted (CIPI/213006.45).

### Measures

#### Sociodemographic characteristics

Participants reported their gender, age range, marital status, number of children, studies completed, employment situation, type of working day and with whom they reside. In addition, we assessed the activity in relevant social groups and satisfaction with that activity. Finally, participants reported their satisfaction with their life partner and with their relationships with work/study colleagues. All questions in the questionnaire were “multiple choice” questions, where participants could select only one of the answer options. The questions and response options are provided in Supplementary Table S1.

#### Physical activity and body awareness activities

Additionally, the participants were asked to know if they performed the physical activity levels recommended by the World Health Organisation, and if they practised body awareness activities (Supplementary Table S1).

#### Sensory Processing Sensitivity

SPS was assessed using the 27-item Spanish version of the Highly Sensitive Person Scale, HSPS-S (Chacón et al., 2021). All items were rated on a 7-point Likert scale ranging from 1 (strongly disagree) to 7 (strongly agree). HSPS-S is a five-factor scale with the following dimensions: sensitivity to overstimulation (SOS), aesthetic sensitivity (AES), low sensory threshold (LST), fine psychophysiological discrimination (FPD) and harm avoidance (HA). Example items are: “Are you badly affected by having a lot to do in a short time?” (SOS), “Are you deeply touched by the visual arts or music?” (AES), “Are you disturbed by intense stimuli, such as loud noises or chaotic scenes?” (LST), “Do you tend to be more sensitive to pain?” (FPD), “Do you give high priority to organising your life to avoid disturbing or overwhelming situations?” (HA). HSPS-S showed a good internal consistence with global Cronbach's α of 0.92 [0.86 (SOS), 0.79 (AES), 0.82 (LST), 0.56 (FPD), 0.67 (HA)].

According to Lionetti et al. (2018), the participants were classified within three sensitivity levels using a percentile criterion (30% low-SPS, 40% medium-SPS and 30% high-SPS). Thus, the cut-off scores established in our sample were 138 (low-SPS to medium-SPS) and 164 (medium-SPS to high-SPS). Subsequently, for analyses based on sensitivity levels, each sensitivity level included only participants whose HSPS score fell within the mean ± standard deviation of HSPS for that sensitivity level (Supplementary Table S2). Additionally, total scores and factors of the HSPS-S across different categories of each of the sociodemographic variables are reported in Supplementary Tables S3–S16.

### Data analysis

Firstly, our interest was to study the impact of sociodemographic variables on predicting high-SPS. Thus, a binary logistic regression analysis was conducted on the criterion variable 'high sensitivity', with the prediction of high sensitivity (1). The analysis was carried out following a stepwise strategy in parameter estimation for regression with the R package GLM (Friedman et al., 2010).

Secondly, the interaction between the sensitivity levels and relevant sociodemographic variables was investigated. For this, robust ANOVAs were computed for each sociodemographic variable and each SPS factor. Subsequently, post hoc analyses were conducted for significant interactions. The Hochberg method was used to correct for multiple comparisons. Partial eta-squared ($\eta_{\text{p}}^{2}$) and d-Cohen were used to measure the effect size of the interactions and of the mean differences, respectively.

## Results

### Sociodemographic predictors of high Sensory Processing Sensitivity

A binary logistic regression analysis was conducted to predict high sensitivity, testing the impact of demographic variables, physical and body awareness activities, as well as personal relationship quality at three levels: social groups, work/study colleagues and life partners. Data showed that demographic variable categories were significant in the logistic analysis predicting high sensitivity. At the same time, wellbeing as well as categories of variables related to social environment were significant predicting high sensitivity.

The odds ratio (OR) values showed interesting insights regarding high-SPS based on demographic variables. Regarding gender, as shown in the literature, being female increases the probability of being a highly sensitive person by 3.61 [Z = 16.29, OR = 3.61]. Similarly, concerning age, OR values increase with age, with the 45-54 range showing the highest OR value predicting high-SPS [Z = 15.02, OR = 7.07]. In terms of marital status, the different categories of the variable significantly predict high-SPS compared to the reference category (single). The conclusion drawn is that high-SPS individuals tend to be either in a relationship [Z = 5.37, OR = 1.73] or married [Z = 4.69, OR = 1.70]. However, within the divorced group, the highest percentage of divorces occurred among high-SPS individuals, making the divorced category [Z = 4.40, OR = 1.84] attain the highest odds ratio. Interestingly, living independently, whether renting or owning, significantly predicts high-SPS [Z = 3.79, OR = 1.43]. Regarding social variables, the variable with the greatest impact in predicting high-SPS was the number of social groups (between 1 and 3 groups), which proved significant [Z = 2.66, OR = 1.24]. Finally, among wellbeing variables, body awareness activities significantly predicted high-SPS [Z = 5.33, OR = 1.44] (Table 1).

**Table 1:** Variables predicting high-SPS.

**Sociodemographic variable: reference category**		**Estimate**	**Sd. error**	**Z value**	**Exp**	**97.5% CI**	
	(Intercept)	−3.16	0.24	−13.20^***^	0.04	0.03	0.07
Gender: male	Female	1.28	0.08	16.29^***^	3.61	3.10	4.22
Age: 18–24 years	25–34 years	0.79	0.10	7.57^***^	2.21	1.80	2.71
	35–44 years	1.43	0.12	12.11^***^	4.17	3.31	5.25
	45–54 years	1.95	0.13	15.02^***^	7.03	5.46	9.08
	55–64 years	1.78	0.17	10.61^***^	5.93	4.27	8.24
Civil status: single	Living as a couple	0.55	0.10	5.37^***^	1.73	1.42	2.12
	Married	0.53	0.11	4.69^***^	1.70	1.37	2.13
	Separated	0.16	0.18	0.86	1.17	0.82	1.66
	Divorced	0.61	0.14	4.40^***^	1.84	1.40	2.42
	Widowed	−0.04	0.35	−0.11	0.96	0.47	1.91
	Other	0.54	0.16	3.32^***^	1.71	1,25	2.35
Number of children: no children	1 child	−0.48	0.10	−5.06^***^	0.62	0.51	0.74
	2 children	−0.51	0.10	−4.98^***^	0.60	0.49	0.73
	≥3 children	−0.75	0.15	−4.94^***^	0.47	0.35	0.63
Educational level: primary	Secondary	−0.14	0.11	−1.28	0.87	0.70	1.08
	Professional training	0.11	0.11	0.93	1.11	0.89	1.39
	University degree	−0.19	0.11	−1.69	0.83	0.67	1.03
	Master or PhD	−0.12	0.12	−0.99	0.89	0.70	1.13
Employment: unemployed	Employed	0.01	0.11	0.07	1.01	0.81	1.25
Work dedication: not working	Partial term	−0.00	0.13	−0.01	1.00	0.78	1.28
	Complete term	−0.12	0.12	−0.98	0.89	0.70	1.12
	Other Term	−0.07	0.15	−0.50	0.93	0.69	1.24
Residence: parents/tutors	Share house	0.16	0.11	1.44	1.17	0.94	1.45
	House rent/property	0.36	0.09	3.79^***^	1.43	1.19	1.72
Physical activity: no	Yes	−0.02	0.06	−0.30	0.98	0.87	1.11
Body awareness activity: no	Yes	0.36	0.07	5.33^***^	1.44	1.26	1.64
Number social groups: None	1–3 groups	0.21	0.08	2.66^**^	1.24	1.06	1.45
	4–6 groups	0.19	0.10	1.91	1.22	0.99	1.48
	7–9 groups	−0.17	0.18	−0.94	0.84	0.58	1.20
	≥10 groups	−0.29	0.21	−1.39	0.75	0.49	1.12
Satisfaction with social groups: poor	Not bad	0.11	0.22	0.49	1.11	0.73	1.71
	Neutral	0.07	0.20	0.35	1.07	0.73	1.61
	Good	−0.10	0.20	−0.48	0.91	0.61	1.36
	Very good	−0.07	0.21	−0.31	0.94	0.62	1.43
Satisfaction with partner: without partner	Poor	−0.36	0.16	−2.17^*^	0.70	0.52	0.99
	Not bad	−0.30	0.12	−2.59^**^	0.74	0.60	0.95
	Neutral	−0.40	0.13	−3.02^**^	0.67	0.52	0.87
	Good	−0.16	0.10	−1.65	0.86	0.71	1.03
	Very good	0.02	0.10	0.20	1.02	0.83	1.25
Satisfaction with work/study colleagues: Without work/study colleagues	Poor	0.39	0.22	1.79	1.48	0.96	2.26
	Not bad	0.02	0.14	0.14	1.02	0.78	1.34
	Neutral	−0.13	0.13	−1.06	0.87	0.68	1.12
	Good	−0.17	0.12	−1.47	0.84	0.67	1.06
	Very good	−0.26	0.13	−1.93	0.77	0.60	1.00

Categories in the table were all significant at 0.05 (^*^), 0.01 (^**^) or 0.001 (^***^) level. Estimate (β coefficient): log-odds coefficient from the logistic regression, Exp(B): exponentiated coefficient (odds ratio). Positive and negative values of Estimate, i.e., Exp > 1 (Exp < 1) for a given sociodemographic category, indicate increased (decreased) probability of high sensitivity in that category compared to the reference category.

### Interaction between sensitivity levels and sociodemographic variables

In this section we investigate the possible interaction between sensitivity levels and sociodemographic variables for each SPS factor and explore that interaction. The sociodemographic variables considered were demographic variables (gender, age) and social variables (number of social groups and satisfaction with partner) that significantly contributed to the prediction of high-SPS. We also included “education level” due to its importance as a demographic variable, and “experience within social group”, in order to look into nature of social groups, since the number of social groups was significant in the logistic regression. Robust ANOVAs showed significant interaction effects between all these variables and the sensitivity levels (low, medium, high) for all SPS subscales (*p* < 0.001), except for gender, for which the interaction was not a significant factor (*p* > 0.05 for all SPS factors). Tables 2–6 report the results of the post hoc analysis conducted on SPS factors for which significant interaction effects between sensitivity levels and the corresponding sociodemographic variable were found. Specific interaction effects found are presented below.

**Table 2:** *Post hoc* analysis on significant interactions between SPS levels and age.

**Age (years)**							
		**18–24** ^a^	**25–34** ^b^	**35–44** ^c^	**45–54** ^d^	**55–64** ^e^	**Interactions (post hoc)**
SOS	Low	4.53 ± 1.11	4.13 ± 1.17	3.93 ± 1.16	3.98 ± 1.12	3.76 ± 0.98	**a:(b**^**s**^**,c**^**m**^**,d**^**m**^**,e**^**h**^**) & b:c**^**s**^ **(*****p*** **<** **0.001); b:e**^**m**^ **(*****p*** **=** **0.003)**; b:d (*p* = 0.083); d:e (*p* = 0.204); c:e (*p* = 0.278); c:d (*p* = 0.443)
	Med	6.06 ± 0.51	5.88 ± 0.49	5.80 ± 0.50	5.72 ± 0.51	5.61 ± 0.48	**a:(b**^**s**^**,c**^**s**^**,d**^**s**^**,e**^**m**^**) (*****p*** **<** **0.001); b:e**^**s**^ **(*****p*** **=** **0.005); b:d**^**s**^ **(*****p*** **=** **0.015)**; c:e (*p* = 0.086); (b,d):c & d:e (*p* = 0.194)
	High	6.69 ± 0.28	6.60 ± 0.33	6.62 ± 0.31	6.61 ± 0.31	6.60 ± 0.33	all comparisons (p =0.924)
LST	Low	3.62 ± 1.14	3.67 ± 1.16	3.83 ± 1.20	3.97 ± 1.25	4.14 ± 1.09	**a:(c**^**s**^**,d**^**s**^**,e**^**m**^**) & b:(d**^**s**^**,e**^**m**^**) (*****p*** **<** **.0.001); b:c**^**s**^ **(*****p*** **=** **0.013)**; c:e (*p* = 0.054); c:d (*p* = 0.246); a:b & d:e (*p* = 0.297)
	Med	5.25 ± 0.78	5.45 ± 0.74	5.68 ± 0.68	5.81 ± 0.64	5.97 ± 0.64	**a:(b**^**s**^**,c**^**s**^**,d**^**s**^**,e**^**m**^**) & b:(c**^**s**^**,d**^**s**^**,e**^**m**^**) (*****p*** **<** **0.001); c:e**^**s**^ **(*****p*** **=** **0.004)**; c:d (*p* = 0.081); d:e (*p* = 0.086)
	High	6.38 ± 0.50	6.56 ± 0.42	6.61 ± 0.42	6.73 ± 0.34	6.76 ± 0.32	**a:(c**^**s**^**,d**^**s**^**,e**^**s**^**) (*****p*** **<** **0.001); b:d** ^**s**^ **(*****p*** **=** **0.014); a:b**^**s**^ **(*****p*** **=** **0.020);** b:e (*p* = 0.083); c:d (*p* = 0.085); c:e (*p* = 0.199); b:c (*p* = 0.700); d:e (*p* = 0.712)
AES	Low	4.58 ± 0.90	4.79 ± 0.93	4.93 ± 0.83	4.86 ± 0.93	5.21 ± 0.82	**a:(b**^**s**^**,c**^**s**^**,d**^**s**^**,e**^**h**^**) & b:e**^**m**^ **(*****p*** **<** **0.001); b:c**^**s**^ **& d:e**^**m**^ **(*****p*** **=** **0.005); c:e**^**s**^ **(*****p*** **=** **0.016);** (b,c):d (*p* = 0.284)
	Med	5.57 ± 0.68	5.72 ± 0.63	5.75 ± 0.62	5.88 ± 0.59	6.00 ± 0.53	**a:(b**^**s**^**,c**^**s**^**,d**^**s**^**,e**^**s**^**) & b:e**^**s**^ **(*****p*** **<** **0.001); b:d**^**s**^ **(*****p*** **=** **0.002); c:e**^**s**^ **(*****p*** **=** **0.003); c:d**^**s**^ **(*****p*** **=** **0.019)**; d:e (*p* = 0.280); b:c (*p* = 0.447)
	High	6.41 ± 0.41	6.49 ± 0.41	6.56 ± 0.38	6.61 ± 0.36	6.60 ± 0.34	**a:d**^**s**^ **(*****p*** **=** **0.001); a:c**^**s**^ **(*****p*** **=** **0.022); b:d**^**s**^ **(*****p*** **=** **0.047);** a:e (*p* = 0.054); b:(c,e) () (p=*p* = 0.398); a:b (*p* = 0.647); c:d (*p* = 0.926); (c,d):e (*p* = 0.947)

(i) Low, Med and High: SPS levels; (ii) a to e: categories of sociodemographic variables; (iii) a:(b,c,d,e): interaction of category a with categories b, c, d and e; (iv) superscripts s, m, h: small, medium, and large effect sizes (reference values for d-Cohen: < 0.3, 0.3-0.5 and >0.5, respectively). FPD and HA factors did not significantly predicted igh sensitivity. The table exclusively reports data concerning SPS factors for which the interaction between sensitivity groups and the sociodemographic variable “age” was significant. Statistically significant interactions are highlighted in bold.

**Table 3:** *Post hoc* analysis on significant interactions between SPS levels and educational level.

**Educational level**							
		**Primary** ^a^	**Secondary** ^b^	**Prof. training** ^c^	**Univ. degree** ^d^	**Master/PhD** ^e^	**Interactions (** ***post hoc*** **)**
SOS	Low	4.64 ± 1.00	4.38 ± 1.20	4.27 ± 1.14	3.97 ± 10.49	4.02 ± 1.03	**a:(b**^**s**^**,c**^**s**^**,d**^**m**^**,e**^**m**^**) & b:(d**^**s**^**,e**^**s**^**) & c:(d**^**s**^**,e**^**s**^**) (*****p*** **<** **0.001); b:c**^**s**^ **(*****p*** **=** **0.034)**; d:e (*p* = 0.458)
	Med	6.02 ± 0.53	6.02 ± 0.50	5.88 ± 0.51	5.79 ± 0.49	5.73 ± 0.52	**a:(d**^**s**^**,e**^**s**^**) & b:(d**^**s**^**,e**^**s**^**) (*****p*** **<** **0.001); b:c**^**s**^ **(*****p*** **=** **0.021); c:e**^**s**^ **(*****p*** **=** **0.030)**; (a,b):c (*p* = 0.070); c:d (*p* = 0.214); d:e (*p* = 0.427); a:b (*p* = 0.977)
	High	6.68 ± 0.30	6.67 ± 0.28	6.63 ± 0.31	6.60 ± 0.33	6.57 ± 0.33	b:e (*p* = 0.607); a:e (*p* = 0.807); other comparisons (*p* = 0.873)
AES	Low	4.46 ± 0.93	4.66 ± 0.92	4.78 ± 0.88	4.87 ± 0.88	4.92 ± 0.88	**a:(b**^**s**^**,c**^**s**^**,d**^**s**^**,e**^**s**^**) & b:(d**^**s**^**,e**^**s**^**) (*****p*** **<** **0.001); b:c**^**s**^ **(*****p*** **=** **0.036)**; c:e (*p* = 0.055); c:d (*p* = 0.094); d:e (*p* = 0.395)
	Med	5.58 ± 0.66	5.60 ± 0.68	5.67 ± 0.63	5.81 ± 0.61	5.85 ± 0.61	**a:(d**^**s**^**,e**^**s**^**) & b:(d**^**s**^**,e**^**s**^**) (*****p*** **<** **0.001); c:e**^**s**^ **(*****p*** **=** **0.002); c:d**^**s**^ **(*****p*** **=** **0.010)**; (a,b):c (*p* = 0.338); a:b & d:e (*p* = 0.679)
	High	6.49 ± 0.41	6.51 ± 0.37	6.51 ± 0.38	6.57 ± 0.40	6.57 ± 0.37	all comparisons (*p* = 0.917)
HA	Low	4.70 ± 1.09	5.01 ± 1.09	5.07 ± 1.14	5.19 ± 1.07	5.15 ± 1.12	**a:(b**^**s**^**,c**^**s**^**,d**^**s**^**,e**^**s**^**) & b:d**^**s**^ **(*****p*** **<** **0.001)**; b:e & c:d (*p* = 0.149); (b,e):c & d:e (*p* = 0.545)
	Med	5.75 ± 0.79	5.78 ± 0.83	5.82 ± 0.36	5.94 ± 0.70	6.00 ± 0.68	**b:e**^**s**^ **(*****p*** **<** **0.001); a:e**^**s**^ **(*****p*** **=** **0.001); b:d**^**s**^ **(*****p*** **=** **0.006); a:d**^**s**^ **(*****p*** **=** **0.011); c:e**^**s**^ **(*****p*** **=** **0.018);** a:(b,c) & b:c & d:e (*p* = 0.606); c:d (*p* = 0.149)
	High	6.55 ± 0.52	6.53 ± 0.53	6.55 ± 0.49	6.61 ± 0.45	6.63 ± 0.45	All comparisons (*p* = 0.983)

(i) Low, Med and High: SPS levels; (ii) a to e: categories of sociodemographic variables; (iii) a:(b,c,d,e): interaction of category a with categories b, c, d and e; (iv) superscripts s, m, h: small, medium, and large effect sizes (reference values for d-Cohen: < 0.3, 0.3–0.5 and > 0.5, respectively). The table exclusively reports data concerning SPS factors for which the interaction between sensitivity groups and the sociodemographic variable 'educational level' was significant. Statistically significant interactions are highlighted in bold.

**Table 4:** *Post hoc* analysis on significant interactions between SPS levels and number of social groups.

**Number of social groups**				
		**1–3**	**None**	**Interactions (** ***post hoc*** **)**
SOS	Low	4.23 ± 1.14	4.51 ± 1.18	***p*** **<** **0.001**^**s**^
	Med	5.88 ± 0.51	6.04 ± 0.51	***p*** **<** **0.001**^**s**^
	High	6.62 ± 0.31	6.69 ± 0.30	*p* = 0.185
AES	Low	4.73 ± 0.87	4.44 ± 0.98	***p*** **<** **0.001**^**s**^
	Med	5.72 ± 0.63	5.51 ± 0.70	***p*** **<** **0.001**^**s**^
	High	6.53 ± 0.39	6.50 ± 0.43	*p* = 0.449
HA	Low	5.08 ± 1.07	4.87 ± 1.19	***p*** **<** **0.001**^**s**^
	Med	5.88 ± 0.73	5.77 ± 0.80	***p*** **=** **0.017**^**s**^
	High	6.58 ± 0.47	6.60 ± 0.49	*p* = 0.595

(i) Low, Med and High: SPS levels; (ii) superscript s indicates small effect size (reference value for d-Cohen: < 0.3). The table exclusively reports data concerning SPS factors for which the interaction between sensitivity groups and the sociodemographic variable “number of social groups” was significant. Statistically significant interactions are highlighted in bold.

**Table 5:** *Post hoc* analysis on significant interactions between SPS levels and experience within social groups.

**Experience within social groups**							
		**Poor** ^a^	**Not bad** ^b^	**Neutral** ^c^	**Good** ^d^	**Very good** ^e^	**Interactions (** ***post hoc*** **)**
SOS	Low	5.25 ± 0.93	4.71 ± 1.10	4.67 ± 0.93	4.31 ± 1.01	3.68 ± 1.30	**a:(b**^**h**^**,c**^**h**^**,d**^**h**^**,e**^**h**^**) & b:(d**^**m**^**,e**^**h**^**) & c:(d**^**s**^**,e**^**h**^**) & d:e**^**s**^ **(*****p*** **<** **0.001);** b:c (*p* = 0.505)
	Med	6.22 ± 0.47	6.11 ± 0.50	5.94 ± 0.51	5.86 ± 0.50	5.74 ± 0.54	**(a**^**h**^**,c**^**s**^**):e & b:(d**^**s**^**,e**^**s**^**) (*****p*** **<** **0.001); a:d**^**m**^ **(*****p*** **=** **0.002); a:c**^**m**^ **& b:c**^**s**^ **& d:e**^**s**^ **(*****p*** **=** **0.022)**; c:d (*p* = 0.086); a:b (*p* = 0.315)
	High	6.75 ± 0.35	6.71 ± 0.26	6.65 ± 0.29	6.59 ± 0.32	6.59 ± 0.32	b:d (***p*** **=** 0.480); b:e (*p* = 0.533); other comparisons (*p* = 0.929)
LST	Low	3.85 ± 1.19	3.99 ± 1.14	3.90 ± 1.10	3.70 ± 1.11	3.47 ± 1.26	**b:(d**^**s**^**,e**^**h**^**) & d:(c**^**s**^**,e**^**s**^**) & c:e**^**h**^ **(*****p*** **<** **0.001); a:e**^**s**^ **(*****p*** **=** **0.004)**; a:(b,d) & b:c (*p* = 0.569); a:c (*p* = 0.650)
	Med	5.71 ± 0.77	5.48 ± 0.82	5.49 ± 0.75	5.52 ± 0.73	5.48 ± 0.79	a:(b,c,e) (*p* = 0.518); a:d (*p* = 0.727); other comparisons (*p* = 0.917)
	High	6.67 ± 0.38	6.60 ± 0.45	3.60 ± 0.45	6.62 ± 0.40	6.60 ± 0.42	all comparisons (*p* = 0.973)
AES	Low	3.98 ± 1.22	4.36 ± 0.91	4.54 ± 0.88	4.78 ± 0.85	4.96 ± 0.89	**a:(b**^**m**^**,c**^**m**^**,d**^**h**^**,e**^**h**^**) & b:(d**^**s**^**,e**^**m**^**) & c:(d**^**s**^**,e**^**s**^**) & d:e**^**s**^ **(*****p*** **<** **0.001); b:c**^**s**^ **(*****p*** **=** **0.003)**
	Med	5.17 ± 0.79	5.45 ± 0.71	5.63 ± 0.66	5.76 ± 0.60	5.89 ± 0.59	**a:(c**^**m**^**,d**^**m**^**,e**^**h**^**) & b:(d**^**s**^**,e**^**s**^**) & c:e**^**s**^ **(*****p*** **<** **0.001); b:c**^**s**^ **& d:(e**^**s**^**,c**^**s**^**) (*****p*** **=** **0.002); a:b**^**s**^ **(*****p*** **=** **0.005)**
	High	6.53 ± 0.36	6.50 ± 0.46	6.51 ± 0.41	6.53 ± 0.37	6.59 ± 0.35	c:e (***p*** **=** 0.725); other comparisons (***p*** **=** 0.968)
FPD	Low	3.24 ± 1.33	3.72 ± 0.93	3.69 ± 0.99	3.61 ± 1.01	3.27 ± 1.18	**(b**^**s**^**,c**^**s**^**,d**^**s**^**):e (*****p*** **<** **0.001); a:(b**^**s**^**,c**^**s**^**) (*****p*** **=** **0.001); a:d**^**s**^ **(*****p*** **=** **0.006);** (b,c):d (*p* = 0.313); a:e & b:c (*p* = 0.778)
	Med	5.02 ± 0.86	5.01 ± 0.81	5.00 ± 0.82	5.04 ± 0.82	4.97 ± 0.82	all comparisons (***p*** **=** 0.946)
	High	6.15 ± 0.80	6.13 ± 0.63	6.01 ± 0.64	6.11 ± 0.60	6.15 ± 0.61	c:e (*p* = 0.135); c:d (*p* = 0.343); other comparisons (*p* = 0.990)
HA	Low	4.46 ± 1.29	4.66 ± 1.18	4.94 ± 1.06	5.05 ± 1.02	5.22 ± 1.18	**a:(c**^**s**^**,d**^**s**^**,e**^**m**^**) & b:(c**^**s**^**,d**^**s**^**,e**^**s**^**) & (c**^**s**^**,d**^**s**^**):e (*****p*** **<** **0.001); c:d**^**s**^ **(*****p*** **=** **0.022)**; a:b (*p* = 0.124)
	Med	5.72 ± 0.88	5.69 ± 0.87	5.83 ± 0.76	5.89 ± 0.69	5.95 ± 0.76	**b:e**^**s**^ **(*****p*** **=** **0.002); b:d**^**s**^ **(*****p*** **=** **0.015)**; c:e (*p* = 0.183); a:e & b:c (*p* = 0.284); (a,c,e):d (*p* = 0.620); a:c (*p* = 0.704); a:b (*p* = 0.804)
	High	6.60 ± 0.66	6.63 ± 0.46	6.55 ± 0.51	6.57 ± 0.48	6.61 ± 0.45	All comparisons (*p* = 0.941)

(i) Low, Med and High: SPS levels; (ii) a to e: categories of sociodemographic variables; (iii) a:(b,c,d,e): interaction of category a with categories b, c, d and e; (iv) superscripts s, m, h: small, medium, and large effect sizes (reference values for d-Cohen: < 0.3, 0.3–0.5 and > 0.5, respectively). The table exclusively reports data concerning SPS factors for which the interaction between sensitivity groups and the sociodemographic variable 'experience within social groups' was significant. Statistically significant interactions are highlighted in bold.

**Table 6:** *Post hoc* analysis on significant interactions between SPS levels and satisfaction with partner.

**Satisfaction with partner**						
		**No partner** ^a^	**Poor** ^b^	**Not bad** ^c^	**Neutral** ^d^	**Interactions (** ***post hoc*** **)**
HA	Low	4.99 ± 1.08	4.58 ± 1.19	4.82 ± 1.02	4.92 ± 0.97	**a:b**^**s**^ **(*****p*** **<** **0.001); b:d**^**s**^ **(*****p*** **=** **0.019)**; a:c (*p* = 0.071); b:c (*p* = 0.134); d:(a,c) (*p* = 0.358)
	Med	5.83 ± 0.77	5.83 ± 0.88	5.77 ± 0.72	5.81 ± 0.81	All comparisons (*p* = 0.988)
	High	6.55 ± 0.51	6.59 ± 0.50	6.54 ± 0.51	6.54 ± 0.50	All comparisons (*p* = 0.951)

(i) Low, Med and High: SPS levels; (ii) a to d: categories of sociodemographic variables; (iii) a:(b,c,d): interaction of category a with categories b, c and d; (iv) superscripts s, m, h: small, medium, and large effect sizes (reference values for d-Cohen: < 0.3, 0.3-0.5 and > 0.5, respectively). The table exclusively reports data concerning SPS factors for which the interaction between sensitivity groups and the sociodemographic variable “satisfaction with partner” was significant. Statistically significant interactions are highlighted in bold.

#### Demographic variables

##### Age

**Figure 1:**
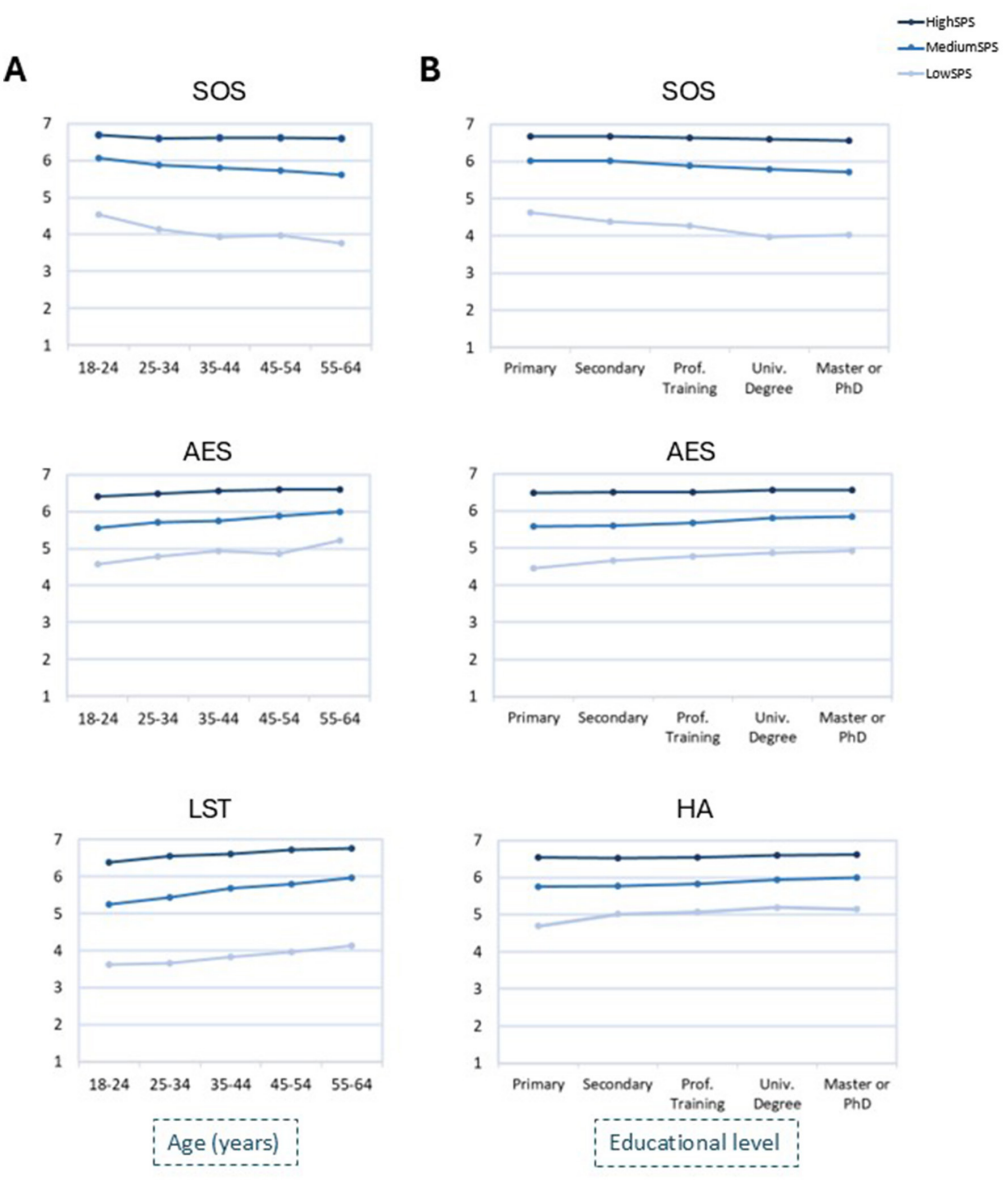
Mean scores of SPS factors, segregated by level of sensitivity (low, medium, high), across categories of the variables “Age” **(A)** and “Educational level” **(B)**. SOS, Sensitivity to Overstimulation; AES, Aesthetic Sensitivity; LST, Low Sensory Threshold; HA, Harm Avoidance.

Significant interaction was found between age and sensitivity levels in SOS, LST (*p* < 0.001, $\eta_{\text{p}}^{2}$ < 0.001) and AES (*p* = 0.001, $\eta_{\text{p}}^{2}$ < 0.001) (Table 2, Figure 1). In general, significant differences with age were found at all SPS levels and they occurred mainly when comparing young (18–24 or 25–34 years) with older ages (35–44, 45–54 or 55–64 years), with older scoring lower in SOS and higher in AES and LST. For high-SPS, SOS did not change with age (*p* = 0.924).

##### Education level

Significant interaction was found in SOS, AES and HA (*p* < 0.001, $\eta_{\text{p}}^{2}$ < 0.001) (Table 3, Figure 1). Low-SPS and medium-SPS presented a significant increase in AES and HA and a decrease in SOS in comparisons between the lowest and highest categories of education level. In contrast, for high-SPS these factors did not change.

#### Social variables

##### Number of social groups

**Figure 2:**
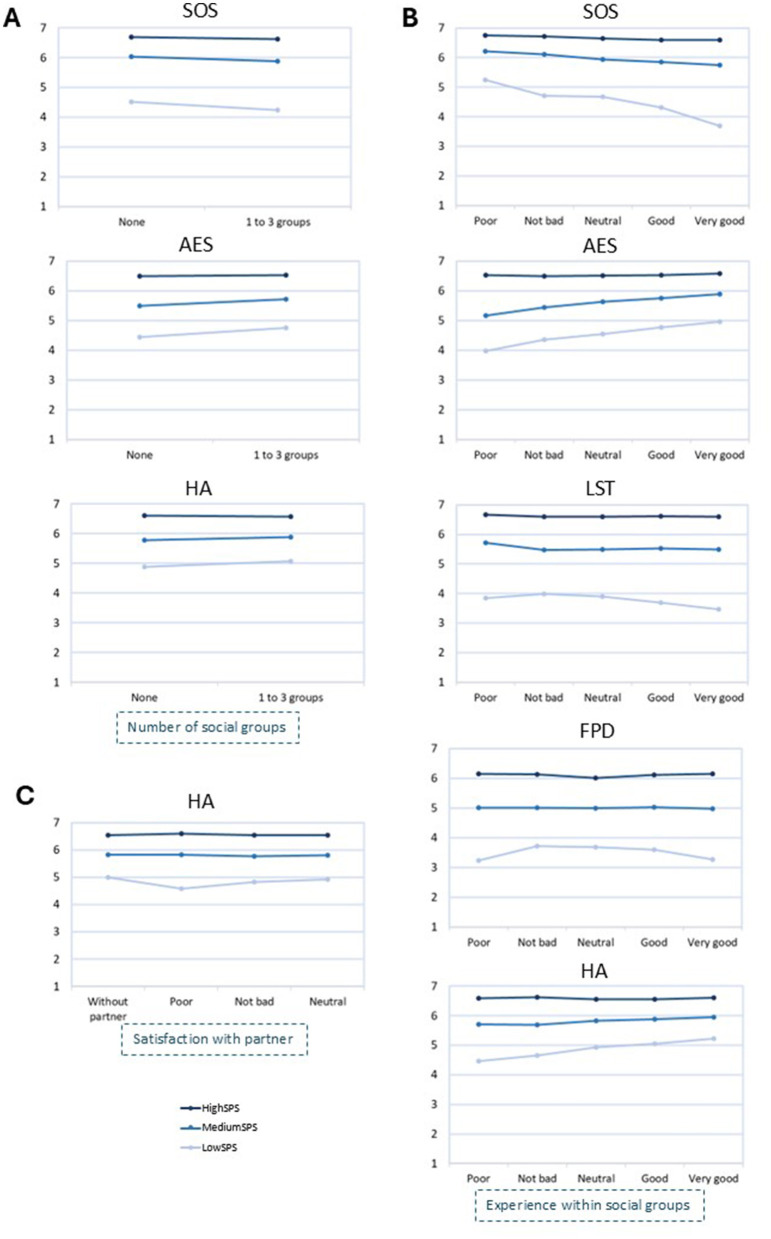
Mean scores of SPS factors, segregated by level of sensitivity (low, medium, high), across categories of the variables “Number of social groups” **(A)**, “Experience within social groups” **(B)** and “Satisfaction with partner” **(C)**. SOS, Sensitivity to Overstimulation; AES, Aesthetic Sensitivity; LST, Low Sensory Threshold; FPD, Fine Psychophysiological Discrimination; HA, Harm Avoidance.

Significant interaction was found for SOS (*p* = 0.001, $\eta_{\text{p}}^{2}$ < 0.001), AES (*p* < 0.001, $\eta_{\text{p}}^{2}$ < 0.001) and HA (*p* = 0.002, $\eta_{\text{p}}^{2}$ < 0.001) (Table 4, Figure 2). Low-SPS and medium-SPS, who participated in between 1 and 3 social groups presented higher AES and HA and lower SOS compared to those who did not participate in any social group. For high-SPS no factors changed significatively.

##### Experience within social groups

Significant interaction was found for all SPS factors (SOS: *p* < 0.001, $\eta_{\text{p}}^{2}$ = 0.002; LST, AES, FPD, HA: *p* < 0.001, $\eta_{\text{p}}^{2}$ < 0.001) (Table 5, Figure 2). For low-SPS and medium-SPS, SOS decreased, and AES and HA increased as experience within social groups improved. For low-SPS, LST and FPD also changed with experience within social groups. For high-SPS no factors changed significatively.

##### Satisfaction with partner

Significant interaction was found between experience in social groups and sensitivity levels in HA (*p* =0.04, $\eta_{\text{p}}^{2}$ < 0.001) (Table 6, Figure 2). Here, only low-SPS experienced changes in HA across levels of satisfaction with partner.

## Discussion

This study primarily sought to answer two key research questions: (1) What sociodemographic variables are important in predicting high-SPS?; and (2) Is the relationship between sociodemographic variables and SPS dependent on the sensitivity level?. By answering these questions, this research describes how SPS and SPS trait manifest at a sociodemographic level, and how the relationship between SPS and sociodemographic variables depends on sensitivity levels.

### Sociodemographic predictors of high Sensory Processing Sensitivity

Previous studies have highlighted the importance of considering the broader social context when exploring sociodemographic factors that characterise individuals (Willroth et al., 2022). To consider this social context, we included the perspective of social relations in a broad sense, as portrayed through the number of social groups, experience in social groups, and satisfaction with partner and colleagues, which, together with the demographic variables, were introduced into a logistic regression model to determine which variables predicted high-SPS. In addition to the aforementioned variables, the model also included the practise of both physical exercise and body awareness activities. These variables were interesting due to their well-known impact on physical and mental health. Specifically, “body awareness activities” predicted high-SPS. This may be attributed to the heightened environmental awareness and aesthetic appreciation often observed in highly sensitive individuals (Aron and Aron, 1997) which is captured by the AES dimension of the SPS (Chacón et al., 2021). However, we cannot rule out that high-SPS individuals need to look for tools, such as body awareness activities, to reduce the level of overstimulation of their nervous system. This can be exemplified by the testimony of a highly sensitive student, who stated: ‘I have not been able to meditate as much as I would like to and manage all the stress that is generated throughout the day; I have somatised it into headaches', within the context of a qualitative study on high sensitivity in a university environment (Morales-Botello et al., 2026).

Among the variables considered in our logistic model, the variable with the greatest impact on the prediction of high-SPS was age, multiplying by 7.03 the probability of high-SPS in the 45- to 54-year-old group compared to the younger group (18–24 years) and with multiplicative factors between 2.21 and 5.93 for the rest of the age ranges. This result highlights the importance of considering this variable in studies on SPS, and its importance within the context of the SPS trait. Previous literature has reported inconsistent findings regarding age-related changes in SPS total scores for adult people (Panagiotidi et al., 2020; Schmitt, 2022; Setti et al., 2022). The present findings may contribute to resolving these inconsistencies in the literature. We found a systematic increase in HSPS score between 18 and 54 years, followed by a modest decline from 55 to 64 years compared to the previous age range (Supplementary Table S4). Several mechanisms may contribute to this observed effect: (i) Development: the maturation or development of individuals may represent the primary basis for the increase in SPS with age, particularly during the transition to adulthood. In contrast, changes associated with 'senescence' may also decelerate or even reverse the age-related rise in SPS; (ii) Environment: exposure to environmental stimuli (including external and internal stimuli such us cognitive/emotional experiences) could enhance Sensory Processing Sensitivity levels; and (iii) Gene-environment interaction: although various genetic components linked to SPS have been identified (Chen et al., 2011; Licht et al., 2020), their interplay with environmental factors may further modulate individual differences in SPS.

The second variable with the greatest impact was gender, where being female multiplied by 3.61 the probability of high-SPS compared to male. Gender differences in SPS have been widely discussed previously. In this sense, our results were contrary to some studies (Chen et al., 2015; Ishibashi et al., 2022), but were in line with most (e.g., Bürger et al., 2024; Chacón et al., 2021; Jentsch et al., 2022; Pluess et al., 2023). Among the causes attributable to such a difference, the main researchers in the field highlight: (i) biological differences and interaction with the environment (Assary et al., 2021); (ii) genetic-age-gender interaction, this mechanism may also contribute, given that previous studies have reported changes with age in sensory thresholds that occur differently between men and women (Leong et al., 2010); (iii) sociocultural differences in emotional expression and sensory perception between men and women, as well as, women's greater propensity to report sensitivity traits due to social norms (Pluess et al., 2018). It is worth noting that, among the possible causes mentioned for the higher scores reported by the female gender, the impact of social norms, such as gender stereotypes regarding sensitivity (Aron and Aron, 1997), may introduce bias, which should also be taken into account when interpreting the results. Subsequent investigations may examine SPS across genders to clarify whether the observed differences reflect biological, psychological, or sociocultural influences.

The different marital status categories multiplied by factors between 1.71 and 1.84 the probability of high sensitivity compared to the singles group. This result could be partially explained in relation to age, since we found a greater proportion of singles in the youngest group, and this group scored lowest in SPS. Alternatively, this might reflect distinct behavioural patterns in relationships. Our data show that highly sensitive individuals had a lower proportion of single people (and higher rates of married/cohabiting individuals), yet notably, nearly twice the rate of divorced/separated individuals compared to other sensitivity groups (Supplementary Table S2). On the other hand, having children (regardless of the number) decreased the probability of high sensitivity, a result that must also be interpreted based on its relationship with age. Nevertheless, to understand the child-SPS levels relationship, further studies that explore it more comprehensively and in a controlled manner would be required.

In terms of residence, independent living significantly heightened the probability of high sensitivity. Similarly, this can be partially explained by age, as with increasing age, individuals are more likely to seek independence, and high sensitivity scores also increased with age. Alternatively, individuals with high sensitivity may prefer living independently, possibly to better cope with their sensitivity.

Finally, educational level did not significantly predict high sensitivity. This finding appears theoretically counterintuitive, as the depth of processing characteristic of high sensitivity (Aron and Aron, 1997) could facilitate complex cognitive tasks typically required in advanced education. However, we should not assume that greater aptitude for certain tasks necessarily translates into preference for undertaking them.

Regarding social variables, satisfaction with partner or work/study colleagues offered an interesting perspective on the SPS trait. Poor/not bad/neutral levels of satisfaction with one's partner decreased the probability of high-SPS. This could reveal that for highly sensitive individuals, the level of satisfaction with their romantic relationship is important, otherwise, they prefer not having a partner. With respect to work/study colleagues' relationship, no category of the variable significantly predicted high sensitivity. However, the “very good satisfaction” category approached significance, with a reduced probability of high-SPS compared to having no work/study colleagues. This finding could also suggest greater social demand.

### Interaction between sensitivity levels and sociodemographic variables

One of the most important results of this work was the greater stability of high-SPS at the sociodemographic level compared with SPS factor scores of individuals without this personality trait (low-SPS and medium-SPS). It is interesting, even paradoxical, that this trait, which is characterised by greater sensitivity to environmental influences (Pluess, 2015; Pluess and Boniwell, 2015), appears “less sensitive” to variation in sociodemographic variables. This has been previously described, although delimited to the cultural context, in a study of neural responses using fMRI (Aron et al., 2010) that revealed that high-SPS individuals presented few cultural differences and low-SPS presented strong differences. This last result is also coherent with our results at a general sociodemographic level. Nevertheless, it is important not to overgeneralise, since increased stability should not be conflated with complete stability, as discussed in contemporary research on personality trait dynamics (Bleidorn et al., 2021).

Next, the main findings regarding the interaction between sensitivity levels and demographic and social variables are discussed, focusing on those SPS factors where the interaction was significant, i.e. where high-SPS can behave differently from the other SPS levels.

#### Demographic variables

A pertinent question that researchers raise is whether SPS is stable throughout development, the answer to which has not yet been fully clarified. Our research revealed increases with age in the AES and LST for all sensitivity levels, decreases in SOS for low-SPS and medium-SPS, and stability of SOS for high-SPS. These results are consistent with those previously reported regarding SOS in adults [Golonka and Gulla, 2021; Licht et al., 2020; Schmitt, 2022 (Study 2)]. However, these studies did not report stability for high-SPS, since they did not distinguish between sensitivity levels. On the other hand, an increased LST with age has been also reported by Licht et al. (2020), although this study also showed decrease in AES. Conversely, findings of Ueno et al. (2019) revealed an age-related decreasing in LST and EOE, and increasing age-related in AES. Differences in sample size, age ranges, and percentage of women could contribute to this discrepancy.

Regarding educational level, previous research has reported contradictory results (Pieroni et al., 2024; Setti et al., 2022), although none reports information at the SPS factors level. In this sense, our work supports the existence of changes in the AES, HA (increase) and SOS (decrease) factors for low-SPS and medium-SPS, while high-SPS did not present variation.

#### Social variables

Interestingly, unlike what happened with low-SPS and medium-SPS, high-SPS was stable for all social variables, and the greatest differences between SPS levels were in participation in social groups (mainly, decreased SOS and increased AES and HA for low-SPS and medium-SPS). This reduction in SOS levels among low or medium-SPS individuals might indicate that within these groups, those more sensitive to overstimulation (higher SOS scores) tend to avoid social groups as a means of self-regulation. Conversely, the results for the highly sensitive group could suggest that they manage overstimulation more effectively. In this line, prior studies have evidenced that despite exhibiting poorer health indicators; highly sensitive individuals exhibited a greater diversity and quantity of coping strategies when faced with highly demanding situations (Morales-Botello et al., 2026). In addition, different effective coping strategies have been described for the three sensitivity groups (Yano et al., 2021).

In the specific context of satisfaction in relationships, we saw that there is no simple relationship between levels of relationship satisfaction and SPS and specifically, for high-SPS, no significant relationship was found. In this context, Zorlular and Uzer (2023) suggested that high-SPS individuals are more likely to experience lower satisfaction in their romantic relationships because they are more vulnerable to the effects of negative emotions and conflicts, although they did not find a significant direct association between SPS and relationship satisfaction.

### Broader implications of the present study

Overall, we found significant interactions between sensitivity levels and most sociodemographic variables only for the SOS, AES and HA factors. This is interesting because it allows a more precise identification of the specific dimensions of SPS trait that make high-SPS individuals more different from others in sociodemographic terms. In addition, this suggests that not all factors of SPS exhibit the same dynamics among individuals, nor are they equally influenced by demographic aspects such as age. In this regard, the understanding of SPS would benefit from future studies that explore its components (factors) using objective measures (e.g. physiological or genetic) in relation to relevant variables such as age or gender, but also including other sociodemographic variables in order to facilitate the connexion between SPS characteristics and their impact or manifestation at the sociodemographic level. These findings further inform ongoing discussions on individual differences in sensitivity and personality. The multidimensional biological model of SPS (Assary et al., 2021) may partially account for the observed differences found at the sociodemographic level in SPS factors across different sensitivity levels. In this context, it is interesting to mention the lower variability of SPS that was observed within high-SPS, which coherently leads towards the greater stability observed in sociodemographic terms across high-SPS individuals and may be relevant within the context of individual differences. Furthermore, our results suggest that care should be taken when studying SPS as a continuum and correlating it with other variables, since evidence of different dynamics depending on SPS levels (low, medium and high) and SPS factors are revealed here.

Secondly, we contextualise this work within the clinical and health framework. From this perspective, studies focused on describing sociodemographic factors associated with certain pathologies are common (Fernández-Alba and Labrador, 2005; Llanos et al., 2015). Knowing such sociodemographic information allows for the identification of particularly vulnerable or at-risk groups. Although the SPS trait is not defined as a pathology, the close relationship between high-SPS and numerous negative clinical aspects frequently found in the literature suggests the need for sociodemographic profiling of the SPS trait. However, to date, no systematic investigation has examined the sociodemographic characteristics associated with SPS. This study addresses this gap by identifying the most likely sociodemographic correlates of high Sensory Processing Sensitivity. Specifically, our findings showed that being female, aged above 25–34 (particularly between 45 and 54), being in a partnered relationship (married, cohabiting, or divorced), and living independently (whether renting or owning) are significant predictors of high sensitivity. Consequently, demographic groups exhibiting these characteristics are more likely to include individuals with high sensitivity personality trait. Such sociodemographic characteristics are expected to hold considerable clinical relevance, facilitating the development of more appropriate psychological treatment with interventions personalised for each profile. Specifically, it could guide the development of targeted strategies for stress regulation and burnout prevention, carefully adapted to individual sensitivity profiles, among other therapeutic applications. The sociodemographic understanding of SPS emerging from this study may also help predict wellbeing-related emotional responses, identifying individuals potentially more susceptible to negative effects in hostile environments or particularly responsive to positive, “flourishing”-conducive settings (Agenor et al., 2017).

In addition, several social variables emerged as significant predictors of high sensitivity. These findings carry multi-level implications. From a scientific standpoint, these variables remain under-researched. The present study provides empirical evidence of the distinct strategies employed by highly sensitive individuals in managing both the quantity and quality of their interpersonal relationships. Consequently, this work establishes a foundation for future research to further examine the emotional and behavioural dimensions of high sensitivity in relational contexts, with potential applications in clinical practise, therapeutic interventions, and self-awareness promotion programmes.

Furthermore, our findings may have practical implications for both educational and occupational settings. Thus, the study of sociodemographic characteristics of SPS can help develop work guidelines for education managers and teachers to create learning environments that particularly benefit highly sensitive individuals. For example, introducing changes in the architecture of schools with quiet spaces, areas where children and young people can take a few minutes without social interaction, and warm lighting. But also, through learning strategies such as working in small groups or introducing creative and multiple sensorial activities. On the other hand, information about different demographic profiles can help create more “friendly” work environments by minimising hostile settings or those that highly sensitive individuals may perceive as aggressive. In this line, previous studies such as Andresen et al. (2018) explained why high-SPS individuals might prefer less stressful environments, including reduced social interaction. The findings showed that highly sensitive people employ avoidance strategies to cope with stressful situations. Consequently, implementing such adaptations in educational and workplace settings could significantly aid in managing overstimulation for this population.

A strategy to amplify the impact of the practical applications of our study involves enhancing the visibility of SPS and the high sensitivity trait. Previous research, using both qualitative and quantitative approaches, has evidenced the importance of trait visibility. Among young adults, visibility appears to mediate greater acceptance of the trait and a reduction in associated difficulties (Morales-Botello et al., 2026). In general, knowledge of the trait seems to contribute to increased self-awareness and better self-management, impacting overall wellbeing (Bas et al., 2021; Lindsay, 2017; Saglietti et al., 2024). The sociodemographic profile provided aids in efficiently targeting campaigns for visibility. This, in turn, supports actions across various domains (policies, clinical practises, educational or work environments) that can contribute to greater dissemination and visibility of this individual difference.

Finally, this study involves a sociodemographic approach to personality traits, specifically the SPS trait. Even though this approach has proven to be interesting, large-scale studies are scarce (Al-Halabí et al., 2010; Butt et al., 2022; Goldberg et al., 2018; Mendlowicz et al., 2000). Therefore, from this perspective, this study could also be considered interesting beyond the boundaries of SPS and could be framed within the broader context of personality traits in general. Furthermore, concordant with an emerging body of research highlighting the potential for social and contextual factors to influence personality changes (Hopwood et al., 2022), the present study evidences that sociodemographic variables, beyond being circumstantial characteristics, can be potentially informative about personality traits. Future studies could investigate the nature or mechanisms underlying the sociodemographic differences in sensitivity groups identified here. This could be pursued through diverse experimental approaches, including genetic, physiological, neuroscientific, and psychological methodologies.

In summary, our findings suggest that advancing knowledge of individual differences in SPS requires future research to extend beyond the use of demographic variables as mere controls, instead examining sociodemographic variables as potentially informative markers of individual differences in SPS.

### Strengths and limitations

To our knowledge, this is the pioneering study examining SPS through a sociodemographic perspective, offering important applications across multiple domains including healthcare, educational settings, and occupational contexts. Moreover, the multi-level sensitivity approach and large sample size represent key strengths of this study. This has enabled the exclusion of participants between sensitivity levels, resulting in more precise profiling of sociodemographic characteristics associated with each SPS level.

However, certain limitations must be considered when interpreting the findings and assessing their generalisability. The generalisation of our findings is mainly limited by two factors. Firstly, it should be noted that the study was conducted exclusively with a Spanish population. Although evidence suggests that the characteristics of high SPS remain consistent across cultures (Aron et al., 2010), further studies will be necessary to replicate our findings in different cultural contexts. Secondly, our sample is predominantly female. While this may initially appear to restrict the generalisation of results, its impact might be limited, given the higher prevalence of high SPS observed in women and their higher scores on the HSP scale. On the other hand, regarding the results of the ANOVAs and post hoc analyses, we must clarify that these analyses did not encompass a complete sociodemographic description of the SPS construct but rather were subject, firstly, to consideration mainly of variables that were significant in predicting high-SPS, and secondly, to the interaction between sensitivity levels and sociodemographic variables. That is, these analyses focused on differences at the sociodemographic level between low, medium and high sensitivity groups. Finally, findings interpretation also conveys the limitations associated with a cross-sectional study.

## Conclusion

The present research provides a descriptive analysis of the sociodemographic aspects associated with Sensory Processing Sensitivity (SPS), encompassing both different sensitivity levels (low-SPS, medium-SPS and high-SPS) and SPS factors perspectives. We evidenced how demographic and social characteristics are significant predictors of high SPS. Furthermore, increased stability of SPS factors was observed under changes in sociodemographic variables when comparing high-SPS with low or medium-SPS individuals. Understanding the sociodemographic characteristics associated with high SPS has important implications across scientific and social domains. Higher SPS scores have been linked to increased vulnerability to adverse environmental factors but also to enhanced responsiveness to positive environmental influences. Access to such sociodemographic data could support early detection and the development of targeted interventions. In clinical practise, this might translate into more effective psychological treatments or preventive approaches (e.g., stress or anxiety reduction techniques tailored to high sensitivity). Similarly, in educational and occupational environments, strategies could be implemented to promote calmer settings that reduce sensory overload while enhancing emotional wellbeing. At the same time, from a more active approach, it could facilitate the adaptation of teaching methods and support in the vocational guidance of high-SPS individuals.

In summary, our study offers knowledge about SPS, and its demographic and social manifestations and we hope it can be helpful in the context of promoting the physical and mental wellbeing of highly sensitive people.

## Data Availability

The datasets presented in this study can be found in online repositories. The names of the repository/repositories and accession number(s) can be found below: (Open Science Framework) https://osf.io/dhnbt/?view_only=af6ed798b5434ac3aa2eda742e6696af.
