# Supplementary material for: Sociodemographic markers of high Sensory Processing Sensitivity: a descriptive study
Source: Front Psychol. 2025 Jul 8;16:1617089. doi: 10.3389/fpsyg.2025.1617089 (PMC12280589; doi:10.3389/fpsyg.2025.1617089)
Supplement: Supplementary file 1 [file Supplementary_file_1.docx]

Supplementary Material

# Supplementary Figures and Tables

## Supplementary tables

**Table S1.** Questionnaire details for sociodemographic characteristics: specific questions and response options

| Question | Response options |
| --- | --- |
| 01 – Indicate your gender: | - Man - Trans Man - Woman - Trans Woman - Non-binary - Others |
| 02 – Indicate your age range: | - From 18 to 24 years - From 25 to 34 years - From 35 to 44 years - From 45 to 54 years - From 55 to 64 years - From 65 to 74 years - Over 74 years |
| 03 – Indicate your marital status: | - Married - Divorced - Separated - Single - I have another marital status - Widowed - I live as a couple |
| 04 – Indicate how many children you have: | - Childless - 1 son/daughter - 2 sons/daughters - 3 or more sons/daughters |
| 05 – Indicates the highest degree studies completed: | - No studies - Primary Studies - Secondary Studies - Intermediate or Higher level of Professional Training - College Degree or Degree - Master's Degree - PhD Studies |
| 06 – What has your employment situation been like in the last 6 months? | - Unemployed - Retired - Active worker |
| 07 – In the case of working, your type of working day is... | - Full time - Part time - I'm not currently working - Another type of working day |
| 08 – Who do you reside with? | - Parents/Tutors - Shared House - Rent/Own House |
| 09 – Do you get at least 150 minutes of moderate aerobic physical activity per week, or 75 minutes of vigorous physical activity? | - Yes - No |
| 10 – Do you regularly practice any type of body awareness activity? (Yoga, Dance, Tai Chi...) | - Yes - No |
| 11 – Indicate the number of social groups (virtual or real) to which you belong, which are significant or important to you (e.g.: sports, artistic groups, virtual groups of family or friends, religious or political communities...) | - None - From 1 to 3 groups - From 4 to 6 groups - From 7 to 9 groups - 10 or more groups |
| 12 – Rate your experience within the most important groups for you: | - Poor - Not good - Neutral - Good - Very good |
| 13 – How satisfactory is your relationship as a couple in general for you? | - I don't have a couple - Poor - Not good - Neutral - Good - Very good |
| 14 – How satisfactory is your relationship with your work/study colleagues for you? | - I don't have work/study colleagues - Poor - Not good - Neutral - Good - Very good |

**Table S2.** Descriptive statistics of the variables segregated into the three SPS levels.

|  | | **HSPS-S** | | |
| --- | --- | --- | --- | --- |
|  |  | **Low (35,6%)** | **Medium (33,8%)** | **High (30,6%)** |
| Gender | Male (28,8%) | 4.16 ± 0.58 (58.8%,47.5%) | 5.63 ± 0.16 (27.9%,23.8%) | 6.50 ± 0.18 (13.3%,12.6%) |
|  | Female (71.2%) | 4.31 ± 0.58 (26.3%,52.5%) | 5.65 ± 0.16 (36.2%,76.2%) | 6.52 ± 0.19 (37.6%,87.4%) |
| Age (years) | 18-24 (32.6%) | 4.27 ± 0.59 (53.1%,48.5%) | 5.62 ± 0.16 (33.6%,32.4%) | 6.46 ± 0.18 (13.3%,14.1%) |
|  | 25-34 (25.2%) | 4.22 ± 0.65 (36.0%,25.4%) | 5.65 ± 0.16 (36.3%,27.0%) | 6.50 ± 0.18 (27.7%,22.8%) |
|  | 35-44 (22.0%) | 4.20 ± 0.62 (26.9%,16.6%) | 5.65 ± 0.16 (32.7%,21.3%) | 6.53 ± 0.18 (40.4%,29.1%) |
|  | 45-54 (15.7%) | 4.20 ± 0.71 (16.3%,7.2%) | 5.68 ± 0.52 (31.1%,14.4%) | 6.55 ± 0.19 (52.6%,27.0%) |
|  | 55-64 (4.6%) | 4.29 ± 0.56 (17.7%,2.3%) | 5.68 ± 0.16 (35.7%,4.9%) | 6.54 ± 0.19 (46.6%,7.0%) |
| Civil status | Single (50.4%) | 4.25 ± 0.62 (45.1%,63.8%) | 5.63 ± 0.16 (34.2%,51.1%) | 6.50 ± 0.19 (20.7%,34.1%) |
|  | Lives as a couple (17.0%) | 4.29 ± 0.59 (24.5%,11.7%) | 5.65 ± 0.16 (36.9%,18.5%) | 6.50 ± 0.17 (38.6%,21.4%) |
|  | Married (20.1%) | 4.18 ± 0.64 (25.7%,4.5%) | 5.66 ± 0.16 (31.5%,18.7%) | 6.55 ± 0.19 (42.8%,28.1%) |
|  | Separated (2.6%) | 4.14 ± 0.68 (31.0%,2,3%) | 5.67 ± 0.16 (33.3%,2.6%) | 6.53 ± 0.19 (35.7%,3.1%) |
|  | Divorced (5.5%) | 4.20 ± 0.70 (23.0%,3.6%) | 5.66 ± 0.15 (26.9%,4.4%) | 6.52 ± 0.20 (50.1%,9.0%) |
|  | Widowed (0.6%) | 4.24 ± 0.44 (27.8%,0.4%) | 5.64 ± 0.11 (30.5%,0.5%) | 6.50 ± 0.17 (41.7%,0.8%) |
|  | Other (3.8%) | 4.37 ± 0.55 (34.5%, 3.7%) | 5.64 ± 0.16 (37.4%,4.2%) | 6.50 ± 0.16 (28.1%,3.5%) |
| Number of children | No Children (68.2%) | 4.27 ± 0.59 (38.3%,73.3%) | 5.64 ± 0.16 (35.3%,71.2%) | 6.50 ± 0.19 (26.4%,59.0%) |
|  | 1 child (13.5%) | 4.21 ± 0.65 (31.1%,11.7%) | 5.67 ± 0.16 (30.4%,12.1%) | 6.53 ± 0.18 (38.5%,16.9%) |
|  | 2 children (14.0%) | 4.15 ± 0.69 (27.0%,10.6%) | 5.67 ± 0.16 (30.9%,12.8%) | 6.55 ± 0.19 (42.1%,19.3%) |
|  | ≥ 3 children (4.3%) | 3.98 ± 0.68 (36.0%,4.4%) | 5.65 ± 0.17 (30.2%,3.9%) | 6.54 ± 0.19 (33.8%,4.8%) |
| Educational level | Primary (12.3%) | 4.25 ± 0.59 (43.1%,14.8%) | 5.63 ± 0.16 (33.5%,12.1%) | 6.53 ± 0.20 (23.4%,9.4%) |
|  | Secondary (27.8%) | 4.25 ± 0.63 (47.3%,37.0%) | 5.64 ± 0.16 (32.3%,26.6%) | 6.50 ± 0.19 (20.4%,18.5%) |
|  | Professional Training (18.0%) | 4.26 ± 0.60 (29.3%,14.9%) | 5.65 ± 0.16 (33.1%,17.7%) | 6.52 ± 0.19 (37.6%,22.3%) |
|  | University degree (27.1%) | 4.20 ± 0.64 (31.4%,23.9%) | 5.65 ± 0.16 (33.7%,27.0%) | 6.52 ± 0.19 (34.9%,30.9%) |
|  | Master or PhD (14.8%) | 4.25 ± 0.58 (22.8%,9.4%) | 5.66 ± 0.16 (38.0%,16.6%) | 6.52 ± 0.18 (39.2%,18.9%) |
| Employment | Unemployed (33.5%) | 4.26 ± 0.59 (43.3%,40.8%) | 5.63 ± 0.17 (32.7%,32.5%) | 6.52 ± 0.19 (24.0%,26.3%) |
|  | Employed (66.5%) | 4.23 ± 0.63 (31.7%,59.2%) | 5.65 ± 0.16 (34.3%,67.5%) | 6.52 ± 0.19 (34.0%,73.7%) |
| Work dedication | Not Working (32.8%) | 4.26 ± 0.59 (42.1%,38.8%) | 5.63 ± 0.17 (33.5%,32.6%) | 6.52 ± 0.18 (24.4%,26.1%) |
|  | Partial Term (14.1%) | 4.30 ± 0.56 (34.6%,13.7%) | 5.65 ± 0.16 (32.7%,13.7%) | 6.52 ± 0.19 (32.7%,15.1%) |
|  | Complete Term (45.7%) | 4.19 ± 0.67 (31.3%,40.0%) | 5.66 ± 0.15 (34.2%,46.2%) | 6.51 ± 0.19 (34.5%,51.6%) |
|  | Other Term (7.4%) | 4.27 ± 0.56 (35.9%,7.5%) | 5.64 ± 0.16 (34.4%,7.5%) | 6.52 ± 0.17 (29.7%,7.2%) |
| Residence | Parents/Tutors (39.6%) | 4.26 ± 0.60 (50.3%,55.9%) | 5.62 ± 0.16 (33.2%,38.9%) | 6.49 ± 0.19 (16,5%,21.4%) |
|  | Share house (11.4%) | 4.24 ± 0.63 (37.6%,12.0%) | 5.65 ± 0.15 (34.2%,11.6%) | 6.51 ± 0.18 (28.2%,10.5%) |
|  | Rent/Own House (49.0%) | 4.21 ± 0.65 (23.3%,32.1%) | 5.66 ± 0.16 (34.2%,49.5%) | 6.53 ± 0.19 (42.5%,68.1%) |
| Physical activity | No (55.9%) | 4.32 ± 0.58 (34.1%,53.6%) | 5.65 ± 0.16 (35.4%,58.6%) | 6.51 ± 0.19 (30.5%,55.7%) |
|  | Yes (44.1%) | 4.15 ± 0.65 (37.5%,46.4%) | 5.64 ± 0.16 (31.7%,41.4%) | 6.52 ± 0.19 (30.8%,44.3%) |
| Body awareness activity | No (76.3%) | 4.26 ± 0.61 (37.8%,80.8%) | 5.64 ± 0.16 (34.2%,77.3%) | 6.51 ± 0.18 (28.0%,69.9%) |
|  | Yes (23.7%) | 4.18 ± 0.65 (28.8%,19.2%) | 5.65 ± 0.17 (32.4%,22.7%) | 6.54 ± 0.19 (38.8%,30.1%) |
| Number social groups | None (22.3%) | 4.26 ± 0.63 (40.6%,25.5%) | 5.65 ± 0.05 (33.9%,22.4%) | 6.63 ± 0.19 (25.5%,18.7%) |
|  | 1 to 3 groups (55.7%) | 4.25 ± 0.61 (34.1%,53.3%) | 5.64 ± 0.16 (34.0%,56.0%) | 6.52 ± 0.18 (31.9%,58.0%) |
|  | 4 to 6 groups (16.9%) | 4.24 ± 0.60 (33.6%,15.9%) | 5.64 ± 0.16 (33.1%,16.5%) | 6.51 ± 0.19 (33.3%,18.4%) |
|  | 7 to 9 groups (2.7%) | 4.14 ± 0.69 (30.3%,2.3%) | 5.66 ± 0.16 (38.2%,3.1%) | 6.50 ± 0.20 (31.5%,2.8%) |
|  | ≥ 10 groups (2.4%) | 4.07 ± 0.65 (44.8%,3.0%) | 5.65 ± 0.16 (27.9%,2.0%) | 6.52 ± 0.19 (27.3%,2.1%) |
| Experience within social groups | Poor (2.5%) | 4.32 ± 0.53 (37.2%,2.6%) | 5.66 ± 0.16 (35.4%,2.6%) | 6.58 ± 0.20 (27.4%,2.3%) |
|  | Not bad (8.3%) | 4.35 ± 0.59 (29.4%,6.9%) | 5.64 ± 0.16 (37.8%,9.3%) | 6.55 ± 0.18 (32.8%,8.9%) |
|  | Neutral (23.3%) | 4.38 ± 0.50 (33.3%,21.8%) | 5.63 ± 0.16 (34.2%,23.6%) | 6.50 ± 0.18 (32.5%,24.7%) |
|  | Good (43.1%) | 4.28 ± 0.57 (34.4%,41.6%) | 5.66 ± 0.16 (35.3%,45.0%) | 6.51 ± 0.19 (30.3%,42.7%) |
|  | Very good (22.8%) | 4.04 ± 0.74 (42.3%,27.1%) | 5.64 ± 0.16 (28.9%,19.5%) | 6.53 ± 0.19 (28.8%,21.4%) |
| Satisfaction with partner | Without partner (37.2%) | 4.29 ± 0.60 (41.4%,43.3%) | 5.64 ± 0.16 (33.2%,36.5%) | 6.50 ± 0.18 (25.4%,30.8%) |
|  | Poor (3.5%) | 4.30 ± 0.52 (31.4%,3.0%) | 5.65 ± 0.16 (33.6%,3.4%) | 6.55 ± 0.17 (35.0%,3.9%) |
|  | Not bad (9.5%) | 4.41 ± 0.52 (27.2%,7.3%) | 5.64 ± 0.17 (38.4%,10.8%) | 6.52 ± 0.17 (34.4%,10.7%) |
|  | Neutral (7.3%) | 4.31 ± 0.57 (37.0%,7.6%) | 5.65 ± 0.15 (35.0%,7.6%) | 6.48 ± 0.20 (28.0%,6.7%) |
|  | Good (23.4%) | 4.25 ± 0.61 (30.4%,19.9%) | 5.65 ± 0.16 (35.5%,24.6%) | 6.53 ± 0.20 (34.1%,26.1%) |
|  | Very good (19.1%) | 4.03 ± 0.71 (35.1%,18.9%) | 5.65 ± 0.16 (30.2%,17.1%) | 6.53 ± 0.19 (34.7%,21.8%) |
| Satisfaction with work/study colleagues | Without work/study colleagues (8.2%) | 4.30 ± 0.55 (32.8%,7.6%) | 5.65 ± 0.15 (31,9%,7.8%) | 6.53 ± 0.19 (35.3%,9.5%) |
|  | Poor (2.0%) | 4.29 ± 0.62 (22.0%,1.3%) | 5.69 ± 0.18 (37.1%,2.2%) | 6.50 ± 0.18 (40.9%,2.7%) |
|  | Not bad (10.6%) | 4.38 ± 0.57 (27.9%,8.3%) | 5.63 ± 0.17 (38.7%,12.1%) | 6.53 ± 0.19 (33.4%,11.6%) |
|  | Neutral (18.2%) | 4.37 ± 0.49 (33.7%,17.2%) | 5.64 ± 0.16 (37.7%,20.3%) | 6.49 ± 0.18 (28.6%,17.1%) |
|  | Good (42.5%) | 4.29 ± 0.58 (35.5%,42.3%) | 5.65 ± 0.16 (34.0%,42.7%) | 6.52 ± 0.18 (30.5%,42.4%) |
|  | Very good (18.5%) | 3.99 ± 0.73 (45.0%,23.3%) | 5.65 ± 0.15 (27.2%,14.9%) | 6.53 ± 0.20 (27.8%,16.7%) |

*Note.* Mean HSPS-S score ± Standard deviation (% of participants in the row category who are in the column's sensitivity level, % of participants in the column's sensitivity level who are in the row's sociodemographic variable category).

| **Table S3.** Descriptive statistics for total scores and factors of the HSPS-S scale across different categories of the sociodemographic variable “Gender” (N = 6507). | | | | | | | | | | | | | | | | | | | | | | | |
| --- | --- | --- | --- | --- | --- | --- | --- | --- | --- | --- | --- | --- | --- | --- | --- | --- | --- | --- | --- | --- | --- | --- | --- |
|  | | | **Gender** | | **HSPS_S** | | **SOS** | | **AES** | | | **LST** | | | **FPD** | | | **HA** | | | |  |  |
| Mean | | | Male | | 4.88 | | 5.00 | | 5.25 | | | 4.44 | | | 4.15 | | | 5.53 | | | |  |  |
|  | | | Female | | 5.63 | | 5.75 | | 5.76 | | | 5.51 | | | 5.08 | | | 5.89 | | | |  |  |
| Standard deviation | | | Male | | 1.03 | | 1.39 | | 1.05 | | | 1.49 | | | 1.36 | | | 1.10 | | | |  |  |
|  | | | Female | | 0.93 | | 1.12 | | 0.96 | | | 1.35 | | | 1.27 | | | 1.00 | | | |  |  |
| **Table S4.** Descriptive statistics for total scores and factors of the HSPS-S scale across different categories of the sociodemographic variable “Age” (N = 6507). | | | | | | | | | | | | | | | | | | | | | | | |
|  | | **Age** | | **HSPS_S** | | | **SOS** | | | **AES** | | | **LST** | | | **FPD** | | | | **HA** | | |  |
| Mean | | 18 to 24 | | 5.01 | | | 5.33 | | | 5.15 | | | 4.53 | | | 4.39 | | | | 5.42 | | |  |
|  | | 25 to 34 | | 5.37 | | | 5.45 | | | 5.59 | | | 5.12 | | | 4.84 | | | | 5.81 | | |  |
|  | | 35 to 44 | | 5.62 | | | 5.63 | | | 5.86 | | | 5.56 | | | 5.03 | | | | 5.99 | | |  |
|  | | 45 to 54 | | 5.89 | | | 5.91 | | | 6.10 | | | 5.99 | | | 5.27 | | | | 6.12 | | |  |
|  | | 55 to 64 | | 5.83 | | | 5.75 | | | 6.14 | | | 6.01 | | | 5.14 | | | | 6.12 | | |  |
| Standard deviation | | 18 to 24 | | 0.95 | | | 1.23 | | | 1.02 | | | 1.41 | | | 1.34 | | | | 1.11 | | |  |
|  | | 25 to 34 | | 1.01 | | | 1.29 | | | 0.98 | | | 1.45 | | | 1.36 | | | | 1.02 | | |  |
|  | | 35 to 44 | | 1.00 | | | 1.29 | | | 0.90 | | | 1.37 | | | 1.33 | | | | 0.94 | | |  |
|  | | 45 to 54 | | 0.90 | | | 1.10 | | | 0.84 | | | 1.18 | | | 1.26 | | | | 0.91 | | |  |
|  | | 55 to 64 | | 0.86 | | | 1.16 | | | 0.73 | | | 1.13 | | | 1.18 | | | | 0.83 | | |  |
| \| **Table S5.** Descriptive statistics for total scores and factors of the HSPS-S scale across different categories of the sociodemographic variable “Civil status” (N = 6507). \| \| \| \| \| \| \| \| \| \| --- \| --- \| --- \| --- \| --- \| --- \| --- \| --- \| --- \| \|  \| **Civil Status** \| **HSPS_S** \| **SOS** \| **AES** \| **LST** \| **FPD** \| **HA** \| \| Mean \| Single \| 5.19 \| 5.39 \| 5.38 \| 4.84 \| 4.56 \| 5.60 \| \|  \| Lives as a couple \| 5.64 \| 5.75 \| 5.79 \| 5.53 \| 5.11 \| 5.93 \| \|  \| Married \| 5.66 \| 5.65 \| 5.89 \| 5.63 \| 5.08 \| 6.05 \| \|  \| Separated \| 5.51 \| 5.52 \| 5.80 \| 5.48 \| 4.86 \| 5.79 \| \|  \| Divorced \| 5.76 \| 5.72 \| 6.06 \| 5.80 \| 5.14 \| 6.03 \| \|  \| Widowed \| 5.63 \| 5.65 \| 5.66 \| 5.64 \| 5.12 \| 6.19 \| \|  \| Other \| 5.44 \| 5.62 \| 5.58 \| 5.19 \| 4.90 \| 5.76 \| \| Standard deviation \| Single \| 1.01 \| 1.28 \| 1.04 \| 1.48 \| 1.37 \| 1.08 \| \|  \| Lives as a couple \| 0.92 \| 1.10 \| 0.92 \| 1.33 \| 1.25 \| 0.95 \| \|  \| Married \| 1.02 \| 1.29 \| 0.91 \| 1.40 \| 1.35 \| 0.94 \| \|  \| Separated \| 1.06 \| 1.32 \| 1.01 \| 1.38 \| 1.39 \| 1.11 \| \|  \| Divorced \| 1.00 \| 1.22 \| 0.87 \| 1.31 \| 1.35 \| 0.94 \| \|  \| Widowed \| 0.97 \| 1.08 \| 1.15 \| 1.27 \| 1.22 \| 0.87 \| \|  \| Other \| 0.92 \| 1.17 \| 1.04 \| 1.34 \| 1.22 \| 1.08 \|   **Table S6.** Descriptive statistics for total scores and factors of the HSPS-S scale across different categories of the sociodemographic variable “Number of children” (N = 6507). | | | | | | | | | | | | | | | | | | | | | | | |
|  | **Number of children** | | | | | **HSPS_S** | | **SOS** | | | **AES** | | | **LST** | | | **FPD** | | **HA** | |  |  |  |
| Mean | No children | | | | | 5.34 | | 5.54 | | | 5.52 | | | 5.05 | | | 4.75 | | 5.71 | |  |  |  |
|  | 1 child | | | | | 5.55 | | 5.55 | | | 5.78 | | | 5.50 | | | 4.97 | | 5.94 | |  |  |  |
|  | 2 children | | | | | 5.63 | | 5.61 | | | 5.89 | | | 5.64 | | | 5.00 | | 6.00 | |  |  |  |
|  | 3 or more children | | | | | 5.35 | | 5.22 | | | 5.72 | | | 5.34 | | | 4.72 | | 5.84 | |  |  |  |
| Standard deviation | No children | | | | | 0.99 | | 1.20 | | | 1.02 | | | 1.47 | | | 1.34 | | 1.06 | |  |  |  |
|  | 1 child | | | | | 1.04 | | 1.32 | | | 0.99 | | | 1.41 | | | 1.36 | | 0.99 | |  |  |  |
|  | 2 children | | | | | 1.05 | | 1.33 | | | 0.93 | | | 1.41 | | | 1.38 | | 0.98 | |  |  |  |
|  | 3 or more children | | | | | 1.17 | | 1.53 | | | 0.99 | | | 1.53 | | | 1.49 | | 1.10 | |  |  |  |

**Table S7.** Descriptive statistics for total scores and factors of the HSPS-S scale across different categories of the sociodemographic variable “Educational level” (N = 6507).

|  | | | | | | | |
| --- | --- | --- | --- | --- | --- | --- | --- |
|  | **Educational Level** | **HSPS_S** | **SOS** | **AES** | **LST** | **FPD** | **HA** |
| Mean | Primary | 5.25 | 5.58 | 5.31 | 4.89 | 4.67 | 5.48 |
|  | Secondary | 5.16 | 5.38 | 5.34 | 4.78 | 4.54 | 5.57 |
|  | Professional Training | 5.57 | 5.69 | 5.73 | 5.45 | 4.99 | 5.87 |
|  | University Degree | 5.50 | 5.50 | 5.78 | 5.38 | 4.89 | 5.94 |
|  | Master/PhD | 5.67 | 5.67 | 5.92 | 5.62 | 5.10 | 6.05 |
| Standard deviation | Primary | 1.01 | 1.13 | 1.10 | 1.50 | 1.39 | 1.15 |
|  | Secondary | 1.02 | 1.31 | 1.04 | 1.50 | 1.37 | 1.09 |
|  | Professional Training | 0.99 | 1.20 | 0.95 | 1.43 | 1.36 | 1.00 |
|  | University Degree | 1.02 | 1.31 | 0.95 | 1.43 | 1.34 | 0.96 |
|  | Master/PhD | 0.92 | 1.15 | 0.87 | 1.29 | 1.24 | 0.93 |

**Table S8.** Descriptive statistics for total scores and factors of the HSPS-S scale across different categories of the sociodemographic variable “Employment” (N = 6507).

|  | | | | | | | |
| --- | --- | --- | --- | --- | --- | --- | --- |
|  | **Employment** | **HSPS_S** | **SOS** | **AES** | **LST** | **FPD** | **HA** |
| Mean | Unemployed | 5.25 | 5.51 | 5.38 | 4.92 | 4.66 | 5.57 |
|  | Employed | 5.49 | 5.55 | 5.73 | 5.35 | 4.89 | 5.90 |
| Standard deviation | Unemployed | 1.01 | 1.22 | 1.07 | 1.47 | 1.37 | 1.13 |
|  | Employed | 1.01 | 1.27 | 0.96 | 1.45 | 1.35 | 0.98 |

**Table S9.** Descriptive statistics for total scores and factors of the HSPS-S scale across different categories of the sociodemographic variable “Work dedication” (N = 6507).

|  | | | | | | | |
| --- | --- | --- | --- | --- | --- | --- | --- |
|  | **Work Dedication** | **HSPS_S** | **SOS** | **AES** | **LST** | **FPD** | **HA** |
| Mean | Not Working | 5.27 | 5.54 | 5.39 | 4.93 | 4.68 | 5.59 |
|  | Partial Term | 5.47 | 5.60 | 5.62 | 5.26 | 4.95 | 5.80 |
|  | Complete Term | 5.50 | 5.52 | 5.76 | 5.39 | 4.88 | 5.93 |
|  | Other Term | 5.41 | 5.52 | 5.69 | 5.16 | 4.80 | 5.73 |
| Standard deviation | Not Working | 1.01 | 1.20 | 1.07 | 1.48 | 1.37 | 1.10 |
|  | Partial Term | 0.99 | 1.18 | 0.99 | 1.44 | 1.32 | 1.05 |
|  | Complete Term | 1.03 | 1.31 | 0.96 | 1.45 | 1.37 | 0.98 |
|  | Other Term | 0.99 | 1.21 | 0.97 | 1.45 | 1.33 | 1.01 |

**Table S10.** Descriptive statistics for total scores and factors of the HSPS-S scale across different categories of the sociodemographic variable “Residence” (N = 6507).

|  | | | | | | | | |
| --- | --- | --- | --- | --- | --- | --- | --- | --- |
|  | **Residence** | **HSPS_S** | **SOS** | **AES** | **LST** | **FPD** | **HA** |  |
| Mean | Parents/Tutor | 5.08 | 5.34 | 5.23 | 4.67 | 4.47 | 5.49 |  |
|  | Share House | 5.36 | 5.45 | 5.60 | 5.13 | 4.83 | 5.72 |  |
|  | Rent/Own House | 5.69 | 5.72 | 5.92 | 5.65 | 5.09 | 6.04 |  |
| Standard deviation | Parents/Tutor | 0.98 | 1.27 | 1.03 | 1.43 | 1.35 | 1.10 |  |
|  | Share House | 1.02 | 1.29 | 0.98 | 1.47 | 1.39 | 1.08 |  |
|  | Rent/Own House | 0.96 | 1.20 | 0.90 | 1.36 | 1.30 | 0.92 |  |

**Table S11.** Descriptive statistics for total scores and factors of the HSPS-S scale across different categories of the sociodemographic variable “Physical activity” (N = 6507).

|  | | | | | | | | |
| --- | --- | --- | --- | --- | --- | --- | --- | --- |
|  | **Physical Activity** | **HSPS_S** | **SOS** | **AES** | **LST** | **FPD** | **HA** |  |
| Mean | No | 5.46 | 5.66 | 5.56 | 5.25 | 4.90 | 5.74 |  |
|  | Yes | 5.35 | 5.38 | 5.68 | 5.14 | 4.71 | 5.84 |  |
| Standard deviation | No | 0.96 | 1.14 | 1.04 | 1.41 | 1.30 | 1.09 |  |
|  | Yes | 1.08 | 1.37 | 0.98 | 1.55 | 1.43 | 0.99 |  |

**Table S12.** Descriptive statistics for total scores and factors of the HSPS-S scale across different categories of the sociodemographic variable “Body awareness activity” (N = 6507).

|  |  |  |  |  |  |  |  |
| --- | --- | --- | --- | --- | --- | --- | --- |
|  | **Body Awareness Activity** | **HSPS_S** | **SOS** | **AES** | **LST** | **FPD** | **HA** |
| Mean | No | 5.36 | 5.52 | 5.53 | 5.13 | 4.77 | 5.74 |
|  | Sí | 5.57 | 5.59 | 5.87 | 5.44 | 4.97 | 5.93 |
| Standard deviation | No | 1.01 | 1.24 | 1.02 | 1.47 | 1.36 | 1.07 |
|  | Sí | 1.03 | 1.29 | 0.94 | 1.46 | 1.37 | 0.95 |

**Table S13.** Descriptive statistics for total scores and factors of the HSPS-S scale across different categories of the sociodemographic variable “Number social groups” (N = 6507).

|  | | | | | | | | |
| --- | --- | --- | --- | --- | --- | --- | --- | --- |
|  | **Number of social groups** | | **HSPS_S** | **SOS** | **AES** | **LST** | **FPD** | **HA** |
| Mean | None |  | 5.31 | 5.58 | 5.33 | 5.09 | 4.70 | 5.62 |
|  | 1 to 3 Groups | | 5.45 | 5.55 | 5.65 | 5.25 | 4.85 | 5.83 |
|  | 4 to 6 Groups | | 5.46 | 5.51 | 5.80 | 5.21 | 4.89 | 5.84 |
|  | 7 to 9 Groups | | 5.46 | 5.28 | 5.94 | 5.34 | 4.86 | 6.05 |
|  | 10 or more Groups | | 5.18 | 5.17 | 5.65 | 4.86 | 4.53 | 5.64 |
| Standard deviation | None |  | 1.02 | 1.23 | 1.13 | 1.46 | 1.37 | 1.15 |
|  | 1 to 3 Groups | | 1.01 | 1.25 | 0.98 | 1.45 | 1.35 | 1.01 |
|  | 4 to 6 Groups | | 1.01 | 1.24 | 0.90 | 1.51 | 1.34 | 1.00 |
|  | 7 to 9 Groups | | 1.03 | 1.38 | 0.89 | 1.45 | 1.39 | 0.82 |
|  | 10 or more Groups | | 1.15 | 1.40 | 0.97 | 1.75 | 1.54 | 1.18 |

**Table S14.** Descriptive statistics for total scores and factors of the HSPS-S scale across different categories of the sociodemographic variable “Experience within social groups” (N = 6507).

|  | | | | | | | |
| --- | --- | --- | --- | --- | --- | --- | --- |
|  | **Experience within social groups** | **HSPS_S** | **SOS** | **AES** | **LST** | **FPD** | **HA** |
| Mean | Poor | 5.41 | 6.00 | 5.10 | 5.28 | 4.67 | 5.49 |
|  | Not bad | 5.56 | 5.90 | 5.47 | 5.41 | 5.00 | 5.69 |
|  | Neutral | 5.50 | 5.75 | 5.55 | 5.32 | 4.89 | 5.76 |
|  | Good | 5.44 | 5.55 | 5.66 | 5.23 | 4.87 | 5.81 |
|  | Very Good | 5.22 | 5.11 | 5.69 | 4.95 | 4.59 | 5.83 |
| Standard deviation | Poor | 0.98 | 0.90 | 1.35 | 1.46 | 1.58 | 1.32 |
|  | Not bad | 0.94 | 1.06 | 1.10 | 1.33 | 1.24 | 1.17 |
|  | Neutral | 0.92 | 1.03 | 1.05 | 1.37 | 1.26 | 1.04 |
|  | Good | 0.98 | 1.17 | 0.91 | 1.44 | 1.31 | 0.99 |
|  | Very Good | 1.18 | 1.56 | 0.92 | 1.64 | 1.54 | 1.07 |

| **Table S15.** Descriptive statistics for total scores and factors of the HSPS-S scale across different categories of the sociodemographic variable “Satisfaction with partner” (N = 6507). | | | | | | | |
| --- | --- | --- | --- | --- | --- | --- | --- |
|  | **Satisfaction with partner** | **HSPS_S** | **SOS** | **AES** | **LST** | **FPD** | **HA** |
| Mean | Without partner | 5.30 | 5.52 | 5.46 | 5.00 | 4.64 | 5.66 |
|  | Poor | 5.54 | 5.77 | 5.55 | 5.39 | 5.05 | 5.70 |
|  | Not bad | 5.61 | 5.80 | 5.73 | 5.50 | 5.02 | 5.77 |
|  | Neutral | 5.40 | 5.58 | 5.53 | 5.18 | 4.82 | 5.69 |
|  | Good | 5.52 | 5.58 | 5.74 | 5.39 | 4.96 | 5.88 |
|  | Very Good | 5.39 | 5.32 | 5.74 | 5.19 | 4.83 | 5.96 |
| Standard deviation | Without partner | 0.99 | 1.19 | 1.08 | 1.47 | 1.33 | 1.07 |
|  | Poor | 0.97 | 1.12 | 1.06 | 1.36 | 1.35 | 1.21 |
|  | Not bad | 0.88 | 1.00 | 0.92 | 1.32 | 1.21 | 1.01 |
|  | Neutral | 0.97 | 1.15 | 1.00 | 1.44 | 1.32 | 1.03 |
|  | Good | 0.99 | 1.21 | 0.95 | 1.40 | 1.33 | 1.00 |
|  | Very Good | 1.15 | 1.53 | 0.96 | 1.60 | 1.50 | 1.00 |

| **Table S16.** Descriptive statistics for total scores and factors of the HSPS-S scale across different categories of the sociodemographic variable “Satisfaction with work/study colleagues” (N = 6507). | | | | | | | | | |
| --- | --- | --- | --- | --- | --- | --- | --- | --- | --- |
|  | **Satisfaction with work/study colleagues** | | **HSPS_S** | | **SOS** | **AES** | **LST** | **FPD** | **HA** |
| Mean | Without work/study colleagues | | 5.52 | | 5.79 | 5.52 | 5.34 | 4.97 | 5.73 |
|  | Poor |  | | 5.71 | 6.20 | 5.55 | 5.53 | 5.16 | 5.63 |
|  | Neutral |  | | 5.46 | 5.73 | 5.52 | 5.22 | 4.84 | 5.73 |
|  | Not bad |  | | 5.58 | 5.89 | 5.58 | 5.41 | 4.99 | 5.74 |
|  | Good |  | | 5.43 | 5.52 | 5.66 | 5.25 | 4.85 | 5.81 |
|  | Very Good | | 5.14 | | 4.99 | 5.67 | 4.86 | 4.51 | 5.86 |
| Standard deviation | Without work/study colleagues | | 0.99 | | 1.14 | 1.15 | 1.42 | 1.38 | 1.12 |
|  | Poor |  | | 0.90 | 0.84 | 1.18 | 1.48 | 1.27 | 1.27 |
|  | Neutral |  | | 0.90 | 0.98 | 1.02 | 1.38 | 1.25 | 1.03 |
|  | Not bad |  | | 0.91 | 1.00 | 1.05 | 1.33 | 1.25 | 1.10 |
|  | Good |  | | 0.99 | 1.19 | 0.96 | 1.44 | 1.32 | 1.00 |
|  | Very Good | | 1.21 | | 1.61 | 1.01 | 1.66 | 1.57 | 1.06 |
